# ERK/GATAD2A/SAA1 axis drives osimertinib resistance via NF-κB and macrophage reprogramming in EGFR-mutated lung adenocarcinoma

**DOI:** 10.1186/s13046-026-03792-6

**Published:** 2026-07-28

**Authors:** Junkan Zhu, Shencheng Ren, Tao Cheng, Guangyao Shan, Han Lin, Xiaolong Huang, Yunyi Bian, Lewei Duan, Yidu Hu, Yingting Wu, Dejun Zeng, Yongqiang Ao, Jiaqi Liang, Shuai Wang, Cheng Zhan

**Affiliations:** 1 Department of Thoracic Surgery, Zhongshan Hospital, Fudan University, No. 180 Feng Lin Road, Shanghai, 200032 China; 2 Department of Thoracic Surgery, Shanghai Chest Hospital, Shanghai Jiao Tong University School of Medicine, Shanghai, China; 3 Cancer Center, Zhongshan Hospital, Fudan University, Shanghai, China; 4 Department of Thoracic Surgery, Zhongshan Hospital, Shanghai Geriatric Medical Center, Fudan University, Shanghai, China

**Keywords:** lung adenocarcinoma, Osimertinib, *SAA1*, Tumor-associated macrophages

## Abstract

**Background:**

Acquired resistance significantly limits the long-term clinical benefit of osimertinib in EGFR-mutant lung adenocarcinoma (LUAD). However, the early non-genetic adaptations and microenvironmental crosstalk that enable initial tumor survival remain poorly defined. This study aimed to elucidate the epigenetic-immune mechanisms driving osimertinib resistance and to identify novel actionable therapeutic targets.

**Methods:**

We integrated transcriptomic profiling and clinical cohort analyses to identify therapy-induced secreted factors during the evolution of EGFR-TKI resistance. The upstream regulatory mechanisms were characterized using ChIP, Co-IP, and dual-luciferase reporter assays. The dual functional roles of the identified factor, serum amyloid A1 (SAA1), in tumor-intrinsic signaling and macrophage polarization were investigated in vitro. Finally, the therapeutic efficacy of combining SAA1 neutralization with osimertinib was evaluated across patient-derived organoids, xenografts, and immunocompetent spontaneous lung tumor models.

**Results:**

We identified SAA1 as a critical therapy-induced secreted factor that progressively increases during the transition from drug tolerance to established resistance, which negatively correlates with clinical outcomes in patients receiving osimertinib. Mechanistically, osimertinib treatment suppressed the ERK-dependent phosphorylation of the transcriptional repressor GATAD2A at Ser114. This event destabilized the NuRD complex, thereby relieving the epigenetic transcriptional repression of SAA1. Secreted SAA1 promoted therapeutic resistance through dual mechanisms: activating the autocrine TLR4/NF-κB survival signaling in tumor cells and inducing the paracrine polarization of pro-tumorigenic SPP1⁺ macrophages. Therapeutically, targeted neutralization of SAA1 synergized robustly with osimertinib to reverse immunosuppressive remodeling and induce profound tumor regression in multiple preclinical models.

**Conclusions:**

Our findings define a novel therapy-induced ERK/GATAD2A/SAA1 epigenetic-immune axis that drives acquired resistance to osimertinib. SAA1 acts as a pivotal bridge between tumor-intrinsic survival adaptations and microenvironmental immune suppression, highlighting SAA1 neutralization as a tractable combinatorial strategy to enhance EGFR-TKI efficacy in LUAD.

**Supplementary Information:**

The online version contains supplementary material available at https://doi.org/10.1186/s13046-026-03792-6.

## Background

Lung adenocarcinoma (LUAD) remains the leading cause of cancer mortality worldwide [1, 2]. While osimertinib is the standard first-line therapy for EGFR-mutant LUAD, acquired resistance inevitably develops within 10–20 months [3–6]. This therapeutic failure is driven by drug-tolerant persister cells (DTPCs) and heterogeneous mechanisms, ranging from EGFR mutations (e.g., C797S) to extensive tumor microenvironment (TME) remodeling [7–10]. Given the limited efficacy of immune checkpoint blockade in this setting, identifying actionable targets to modulate TME-mediated resistance is urgently needed [11].

Secreted factors within the TME are central to acquired resistance. Drug-induced transcriptional reprogramming and TME-derived signals promote the release of cytokines, chemokines, and matrix-associated proteins that activate survival pathways, suppress apoptosis, and foster immune evasion [12, 13]. Although targeting secreted proteins has proven clinically effective in other contexts, the identity of secreted mediators specifically induced during EGFR-TKI treatment and their functional contribution to osimertinib resistance remain unclear [14, 15].

Here, we identify serum amyloid A1 (SAA1) as a key mediator of osimertinib resistance, robustly upregulated in EGFR-mutant LUAD upon treatment. Mechanistically, osimertinib inhibits ERK-dependent phosphorylation of GATAD2A at S114, relieving transcriptional repression of SAA1. Functionally, SAA1 drives resistance via dual mechanisms: autocrine NF-κB activation and paracrine polarization of pro-tumorigenic macrophages. Importantly, combining osimertinib with SAA1 neutralization yields potent therapeutic benefit across multiple in vivo models, providing a compelling rationale for targeting this axis to overcome acquired resistance.

## Results

### Osimertinib upregulates SAA1, which associates with poor clinical outcomes in LUAD

**Fig. 1:**
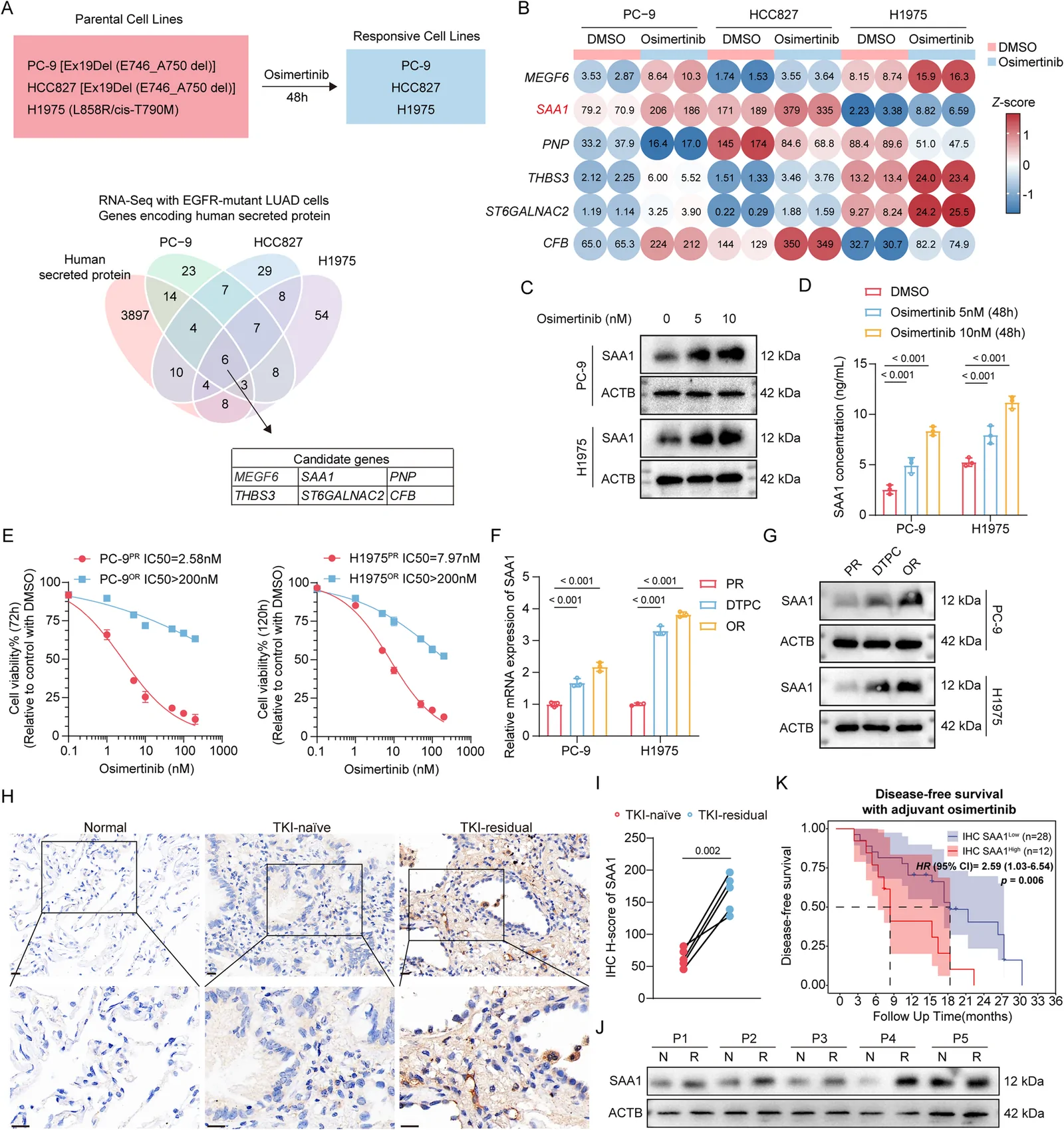
Osimertinib upregulates SAA1, which associates with poor clinical outcomes in LUAD. **A** Schematic of short-term osimertinib exposure (IC_50_, 48 h) in three EGFR-mutant LUAD cell lines (PC-9, HCC827, H1975). Venn diagram illustrates consistently dysregulated secreted-protein genes across the three cell lines. **B** RNA-seq analysis identifying six differentially expressed secreted-protein genes, with FPKM shown in each dot. **C**-**D** Western blot, and ELISA assay validation of SAA1 upregulation, demonstrating a dose-dependent increase in PC-9 and H1975 cells (*n* = 3). **E** Relative cell viability assays confirming ≥ 20-fold resistance to osimertinib in established OR cell lines (PC-9^OR^, H1975^OR^) (*n* = 3). **F**-**G** qPCR and western blot results of SAA1 expression across DTPCs and long-term OR models (*n* = 3). **H** Representative IHC staining of SAA1 in normal lung tissue, LUAD tumors before TKI treatment, and TKI-treated LUAD samples. Scale bar, 20 μm. **I** Quantification of SAA1 IHC H-scores in paired EGFR-mutant LUAD samples collected before and after neoadjuvant osimertinib treatment (*n* = 5). **J** Western blot analysis of paired EGFR-mutant LUAD tumors before (TKI-naïve) and after osimertinib (TKI-residual) treatment (*n* = 5). **K** Kaplan-Meier analysis of disease-free survival of adjuvant osimertinib-treated LUAD patients (*n* = 40). Dunnett’s multiple comparison tests were applied for panels **D** and **F**; two-way ANOVA test for panel E; Paired Student’s t-test for panel I; and log-rank test for panel K. Abbreviations: PR, parental; OR, osimertinib-resistant; DTPC, drug-tolerant persister cells; TKI, tyrosine kinase inhibitor; IHC, immunohistochemistry

To identify secreted mediators of osimertinib resistance, we subjected three EGFR-mutant LUAD cell lines (PC-9, HCC827, and H1975) to osimertinib at respective IC_50_ concentrations for 48 h (Fig. 1A). RNA-seq profiling revealed six differentially expressed genes that encode secreted proteins (*MEGF6*, *SAA1*, *PNP*, *THBS3*, *ST6GALNAC2*, and *CFB*). Among these candidates, SAA1 was prioritized due to its comparatively high basal abundance and robust transcriptional induction following osimertinib exposure (Fig. 1B). Despite its frequent identification as a potential biomarker, the functional significance of SAA1 in LUAD remains largely unexamined [16, 17].

Western blot and Enzyme-linked immunosorbent assay (ELISA) confirmed a dose-dependent increase in SAA1 secretion, with extracellular levels exceeding 10 ng/mL after 10 nM osimertinib exposure (Fig. 1C, D). To determine whether this induction tracked along the resistance continuum, we examined SAA1 expression in short-term drug-tolerant persister cells (DTPCs) (10 days; 200 nM for PC-9, 1 µM for H1975) and in long-term acquired osimertinib-resistant (OR) lines (stepwise dose escalation over three months) [18, 19]. Phenotypically, OR cells exhibited a > 20-fold increase in osimertinib IC_50_ and displayed broad cross-resistance to gefitinib, afatinib, and the fourth-generation inhibitor BDTX-1535 (Fig. 1E; Figure S1). Crucially, SAA1 expression exhibited a distinct, stepwise elevation transitioning from the DTPC state to established resistance, suggesting its pivotal role in sustaining the drug-tolerant phenotype (Fig. 1F, G).

Clinical samples mirrored these findings. Immunohistochemistry demonstrated marked SAA1 protein accumulation in EGFR-mutant LUAD tumors compared to normal tissues, with further enrichment observed in post-treatment residual samples (Fig. 1H). Analysis of five paired EGFR-mutant LUAD samples collected before and after neoadjuvant osimertinib showed increased SAA1 expression in post-treatment residual tumors. IHC H-score quantification demonstrated elevated SAA1 protein levels in TKI-residual samples (Fig. 1I), consistent with increased SAA1 mRNA and protein quantification (Figure S1E). Moreover, in a retrospective cohort of 40 patients receiving adjuvant osimertinib, high baseline SAA1 expression was significantly associated with inferior disease-free survival, conferring an increased risk of recurrence (*HR* = 2.59, *p* = 0.006) (Fig. 1K). Collectively, these multi-tiered data indicate that SAA1 is induced during osimertinib adaptation and resistance, and suggest that elevated baseline SAA1 expression may be associated with unfavorable outcomes in EGFR-mutant LUAD patients receiving adjuvant osimertinib.

### SAA1 is required for both drug tolerance and established osimertinib resistance

**Fig. 2:**
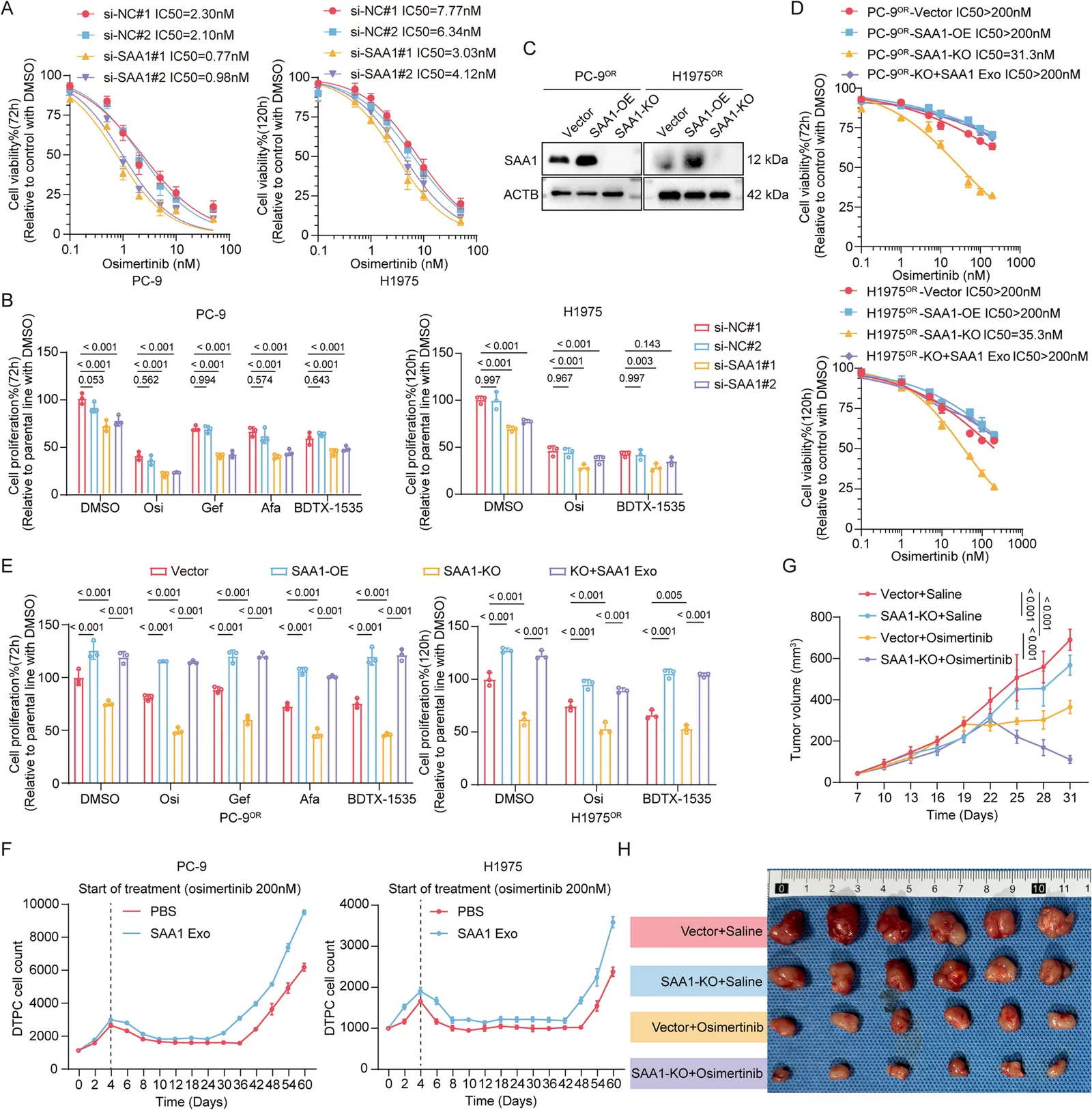
SAA1 is required for both drug tolerance and established osimertinib resistance. **A**-**B** Relative cell viability and proliferation assays with different generations of EGFR-TKI (10 nM) in parental EGFR-mutant LUAD cell lines transfected with two independent siRNAs targeting SAA1 in PC-9 and H1975 cells (*n* = 3). **C** Western blot confirming the establishment of SAA1-OE and SAA1-KO OR cell models generated by lentiviral transduction. **D**-**E** Relative cell viability and proliferation assays (10 nM) with different generations of EGFR-TKI in SAA1-OE, SAA1-KO OR models, and SAA1-KO cells rescued with exogenous SAA1 (100 ng/ml) (*n* = 3). **F** Quantification of DTPC formation efficiency in LUAD cells treated with PBS or exogenous SAA1 (100 ng/ml) (*n* = 3). **G**-**H** In vivo assessment using a PC-9^OR^ CDX model established with cells transduced with either a negative control vector or SAA1-KO lentivirus. Mice received daily oral gavage of osimertinib (5 mg/kg) or saline for 9 consecutive days (*n* = 6). Tumor growth curves (**G**) and representative final tumor specimens (**H**) are shown. Dunnett’s multiple comparison tests were used for panels **B** and **E**, and two-way ANOVA test was applied for panel **G**. Abbreviations: SAA1-OE, SAA1 overexpression; SAA1-KO, SAA1 knockout; OR, osimertinib-resistant; Osi, osimertinib; Gef, gefitinib; Afa, afatinib; TKI, tyrosine kinase inhibitor; DTPC, drug-tolerant persister cells

To delineate the functional requirement of SAA1 in intrinsic resistance, we performed loss-of-function assays using two independent siRNAs. In parental cells, SAA1 knockdown sensitized cells to osimertinib, reducing the IC50 by approximately 3-fold and significantly suppressing proliferation rates (Fig. 2A, B; Supplementary Figure S2A, B).

We subsequently interrogated SAA1 function in established resistance using SAA1-overexpressing (SAA1-OE) and knockout (SAA1-KO) PC-9^OR^ and H1975^OR^ models (Fig. 2C). SAA1 overexpression conferred robust cross-resistance to EGFR-TKIs, whereas CRISPR/Cas9-mediated ablation of SAA1 restored drug sensitivity, which was fully reversible upon exogenous SAA1 supplementation (Fig. 2D, E). Colony formation and EdU assays corroborated that SAA1 is required for clonogenic survival and DNA synthesis under drug pressure (Supplementary Figure S2C-E). Crucially, Annexin V/PI flow cytometry revealed that SAA1 ablation induced massive apoptosis upon TKI treatment and can be rescued by exogenous SAA1 (Supplementary Figure S2F, G).

Given the progressive induction of SAA1 during initial treatment, we hypothesized it is essential for the survival of DTPCs. Supplementation with exogenous SAA1 significantly enhanced DTPC formation efficiency (Fig. 2F). Furthermore, long-term tracking demonstrated that SAA1 ablation profoundly delayed the emergence of DTPCs, whereas SAA1 overexpression accelerated acquired resistance (Supplementary Figure S2H).

Importantly, to clarify whether this dependency is strictly restricted to the resistant state, we evaluated SAA1-KO effects in parallel across parental and OR lines. While SAA1 ablation exerted a moderate growth-suppressive effect in parental cells, it triggered a drastically more profound proliferation blockade and heightened osimertinib sensitivity in OR cells (Supplementary Figure S2I, J).

Finally, we evaluated therapeutic efficacy in vivo using a PC-9^OR^ CDX model. While osimertinib monotherapy yielded modest inhibition, combining SAA1 knockout with osimertinib induced potent tumor regression (~ 100 mm³ vs. ~300 mm³, *p* < 0.001) (Fig. 2G, H). Immunohistochemical analysis in the combination group confirmed not only a marked reduction in the Ki67 proliferation index but also a striking accumulation of cleaved caspase-3 positive cells, indicating profound in vivo apoptotic sensitization (Supplementary Figure S2K). Collectively, these data establish SAA1 as a critical anti-apoptotic driver of both the initial drug-tolerant state and established acquired resistance.

### SAA1 activates the TLR4/NF-κB pathway to drive osimertinib resistance

**Fig. 3:**
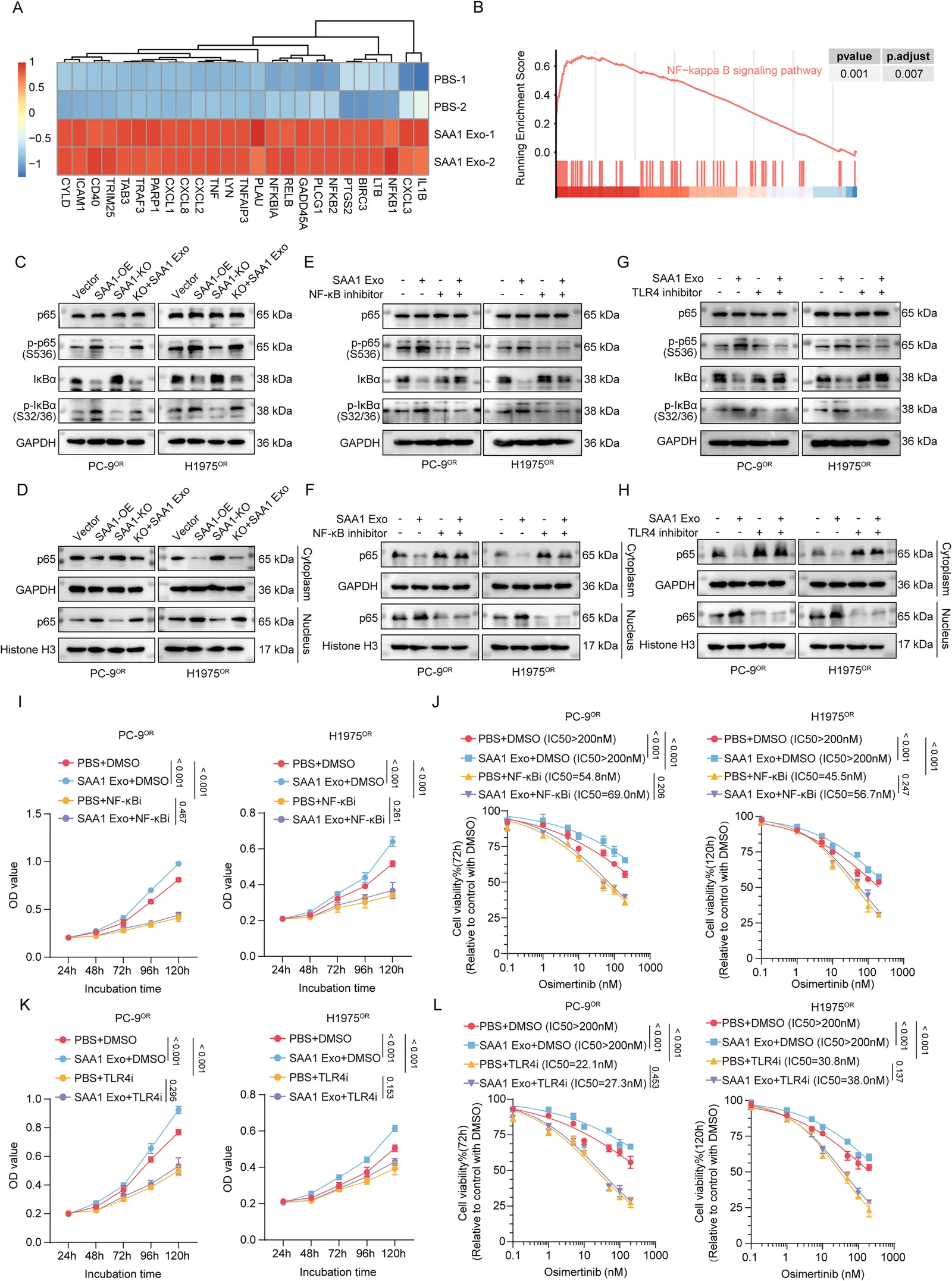
SAA1 activates the TLR4/NF-κB pathway to drive osimertinib resistance. **A** Heatmap showing the expression patterns of NF-κB-associated genes in PC-9 cells treated with exogenous SAA1 (100 ng/ml, 24 h) compared with PBS. **B** GSEA plot indicating enrichment of the NF-κB signaling pathway in SAA1-treated PC-9 cells. **C**-**D** Western blot analysis of whole-cell lysates (**C**, assessing p65, p-p65, IκBα, and p-IκBα) and subcellular fractions (**D**, assessing nuclear and cytoplasmic p65) in PC-9^OR^ and H1975^OR^ cells transfected with vector control, SAA1 overexpression, SAA1 knockout, or SAA1 knockout rescued with exogenous SAA1. **E**-**F** Western blot evaluating the effect of the NF-κB inhibitor BAY 11-7082 (5 µM) on SAA1-induced pathway activation in whole-cell lysates (**E**) and subcellular fractions (**F**). **G**-**H** Western blot evaluating the effect of the TLR4 inhibitor TAK-242 (10 µM) on SAA1-induced pathway activation in whole-cell lysates (**G**) and subcellular fractions (**H**). **I**-**L** Cell proliferation curves (**I**, **K**) and relative cell viability assays determining osimertinib IC50 (**J**, **L**) in PC-9^OR^ and H1975^OR^ cells treated with PBS or exogenous SAA1, in the presence or absence of BAY 11-7082 (5 µM) or TAK-242 (10 µM) (*n* = 3). Two-way ANOVA tests were applied for panels **I**-**L**. Abbreviations: SAA1-OE, SAA1 overexpression; SAA1-KO, SAA1 knockout; OR, osimertinib-resistant; NF-κBi, NF-κB inhibitor; TLR4i, TLR4 inhibitor

To elucidate the mechanism underlying SAA1-mediated resistance, we interrogated signaling pathways associated with high SAA1 expression. Analysis of TCGA-LUAD datasets revealed a significant enrichment of NF-κB signaling in SAA1-high tumors (Supplementary Figure S3A, B). Consistent with these clinical observations, RNA-seq profiling of PC-9 and H1975 cells treated with exogenous SAA1 demonstrated a robust, coordinated upregulation of multiple NF-κB-associated genes (Fig. 3A, B; Supplementary Figure S3C-E).

To rigorously validate this pathway activation across the evolution of resistance, we assessed the specific phosphorylation status and subcellular localization of the NF-κB p65 (RelA) subunit. Biochemically, both DTPCs and established OR lines displayed markedly elevated phosphorylation of p65 at the classical Serine 536 site (p-p65 S536), coupled with increased phosphorylation and acute degradation of IκBα, compared to their parental counterparts (Supplementary Figure S3F). This intense pathway activation was accompanied by profound nuclear translocation of p65 (Supplementary Figure S3G) and a synchronized transcriptional upregulation of canonical downstream targets, including *NFKBIA*, *BCL2*, *CXCL8*, and *IL6* (Supplementary Figure S3H). Importantly, the hyperactivation of this axis was strictly dependent on SAA1. Genetic ablation (SAA1-KO) completely suppressed p-p65 activation and its nuclear accumulation, which was fully rescued by the addition of exogenous SAA1 (Fig. 3C, D; Supplementary Figure S3I).

Because SAA1 functions as a secreted factor, we sought to pinpoint the primary membrane receptor mediating this pro-survival signal. We evaluated TLR2 and TLR4, two prominent candidate receptors implicated in SAA1 signaling. While siRNA-mediated depletion of both receptors was highly efficient, the knockdown of TLR4 sensitized OR cells to osimertinib far more profoundly than TLR2 depletion (Supplementary Figure S4A-C). Subsequent biochemical validation confirmed that silencing TLR4 specifically and comprehensively abrogated SAA1-induced p65 phosphorylation, nuclear translocation, and downstream target gene transcription, firmly establishing TLR4 as the indispensable conduit for SAA1-driven NF-κB activation (Supplementary Figure S4D-G).

Finally, we evaluated the functional and phenotypic consequences of blocking this axis using specific pharmacological inhibitors against NF-κB (BAY 11-7082) or TLR4 (TAK-242). Both inhibitors effectively abolished SAA1-induced p-p65 (S536) activation and nuclear translocation (Fig. 3E-H). Crucially, addressing the classical role of NF-κB in conferring tumor survival, we demonstrated that while blocking this axis impaired cell proliferation to some extent, its paramount mechanism of action was governing apoptotic resistance (Fig. 3I-L). Flow cytometry assays revealed that SAA1 robustly shielded OR cells from osimertinib-induced apoptosis. However, targeted co-treatment with either NF-κB inhibitor or TLR4 inhibitor acutely dismantled this anti-apoptotic shield, fully resensitizing the resistant tumor cells to osimertinib-induced cell death (Supplementary Figure S4H, I). Together, these multidimensional data demonstrate that SAA1 strictly relies on the TLR4/NF-κB survival axis to confer osimertinib resistance.

### SAA1 reshapes the tumor microenvironment by promoting macrophage recruitment and pro-tumorigenic polarization

Secreted proteins not only regulate tumor-intrinsic functions through autocrine signaling but, more importantly, orchestrate TME remodeling via paracrine interactions. SAA1 has previously been identified as a key factor in the invasive zone of liver cancer, where it recruits macrophages via FPR1/TLR receptors to drive M2-like polarization [20].

**Fig. 4:**
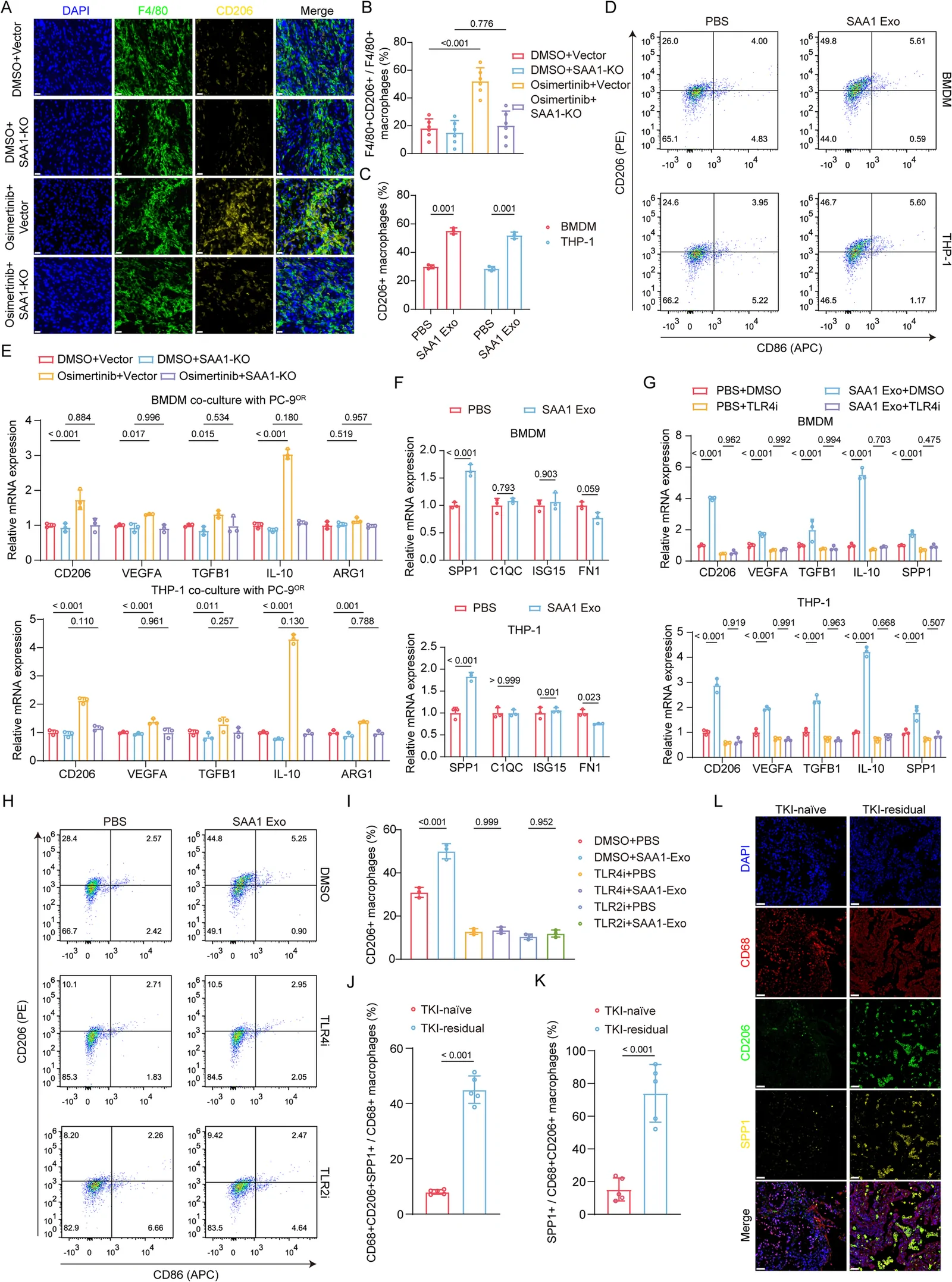
SAA1 reshapes the tumor microenvironment by promoting macrophage recruitment and pro-tumorigenic polarization. **A**-**B** Representative immunofluorescence images (**A**) and corresponding quantification (**B**) of F4/80⁺ (green) and CD206⁺ (yellow) macrophages in tumors from PC-9^OR^ CDX models treated with vehicle or osimertinib, comparing vector control and SAA1-KO groups. Scale bar, 20 μm. **C**-**D** Flow cytometry plots (**D**) and statistical quantification (**C**) of M2 macrophage polarization (CD206⁺) in BMDM and THP-1 cells treated with PBS or exogenous SAA1 (100 ng/ml, 24 h). **E** qPCR analysis of classical M2 macrophage markers (*CD206*, *VEGFA*, *TGFB1*, *IL-10*, *ARG1*) in THP-1 and BMDM cells co-cultured with PC-9^OR^ cell lines under osimertinib treatment, comparing vector control and SAA1 knockout conditions (*n* = 3). **F** qPCR analysis of newly proposed macrophage markers (*SPP1*, *C1QC*, *ISG15*, *FN1*) in THP-1 and BMDM cells after exposure to exogenous SAA1(100 ng/ml, 24 h) (*n* = 3). **G** qPCR analysis of M2 markers and SPP1 in THP-1 and BMDM cells treated with TAK-242 (10 µM, 24 h), with or without exogenous SAA1 (100 ng/ml, 24 h) (*n* = 3). **H**-**I** Flow cytometry analysis (**H**) and comprehensive quantification (**I**) of CD206⁺ macrophages in THP-1 cells treated with PBS or exogenous SAA1 (100 ng/ml, 24 h), combined with DMSO, TAK-242 (10 µM, 24 h), or C29 (50 µM, 24 h), demonstrating the comparative blockade efficacy of distinct TLR pathways (*n* = 3). **J**-**L** Representative multiplex immunofluorescence images (**L**) and quantitative analysis (**J**, **K**) of CD68⁺ (red), CD206⁺ (green), and SPP1⁺ (yellow) macrophage infiltration in paired LUAD patient samples before (TKI-naïve) and after (TKI-residual) osimertinib therapy. Scale bar, 50 μm. Student’s t-tests were for panels **C**, **F**, **J**, **K** and one-way or two-way ANOVA with Dunnett’s multiple comparison tests were for panels B, E, G, I. Abbreviations: OR, osimertinib-resistant; SAA1-KO, SAA1 knockout; Exo, exogenous; TKI, tyrosine kinase inhibitor; IHC, immunohistochemistry

In xenograft tumors treated with osimertinib, we observed a marked accumulation of F4/80⁺CD206⁺ macrophages, a feature that was largely abolished when SAA1 was genetically depleted in tumor cells, as confirmed by rigorous quantitative analysis (Fig. 4A, B). This indicated that tumor-derived SAA1 actively recruits and programs macrophages during therapy. Furthermore, direct treatment with exogenous SAA1 was sufficient to drive M2 polarization, as evidenced by flow cytometry, which showed a significant expansion of the CD206⁺ population from ~ 30% in controls to ~ 50% in SAA1-treated groups (Fig. 4C, D; Figure S5A). To validate this paracrine crosstalk, we co-cultured established human (THP-1) and mouse (BMDM) macrophage models with OR cell lines. Consistent with the in vivo data, exposure to osimertinib-treated tumor cells induced a robust upregulation of classical M2 markers (*CD206*, *VEGFA*, *TGFB1*, *IL-10*, *ARG1*) in both macrophage types, with IL-10 showing a particularly strong induction. This polarization was strictly dependent on tumor-derived SAA1, as SAA1 knockout in the cancer cells significantly reversed marker expression to near-basal levels (Fig. 4E; Figure S5B).

Beyond the classical M1/M2 paradigm, SAA1 also induced strong expression of SPP1, a marker of pro-angiogenic, tumor-supportive macrophage subsets recently implicated in therapy resistance (Fig. 4F) [21]. Mechanistically, we sought to delineate the specific receptor dependency of this macrophage reprogramming. While pharmacological inhibition of TLR4 (TAK-242) partially attenuated the expression of M2 markers and the frequency of CD206⁺ macrophage, head-to-head comparisons revealed that inhibition of TLR2 (inhibited by C29) exerted a more profound suppressive effect (Fig. 4G-I; Figure S5C-E). Treatment with a TLR2 inhibitor nearly completely reversed the SAA1-induced upregulation of M2 markers and CD206⁺ polarization, identifying TLR2 as the predominant paracrine receptor mediating SAA1 signaling in macrophages (Fig. 4H-I; Figure S5C-E). Clinically, multiplex immunofluorescence analysis of the same five paired EGFR-mutant LUAD samples described in Fig. 1H–I further supported these findings. These samples were collected before and after neoadjuvant osimertinib treatment, with TKI-naïve specimens obtained from pre-treatment needle biopsies and TKI-residual specimens obtained from surgically resected residual tumors. Crucially, co-staining revealed that SPP1 was predominantly induced within the CD206⁺ compartment (Fig. 4L). Quantitative analysis demonstrated a striking increase in the proportion of SPP1⁺ cells among CD68⁺CD206⁺ macrophages in TKI-residual tissues compared to TKI-naïve baselines (Fig. 4J, K). These data demonstrate that osimertinib-induced SAA1 actively reshapes the immune microenvironment by driving classical M2 macrophages into a highly specialized, pro-tumorigenic SPP1⁺/CD206⁺ terminal state.

### Osimertinib promotes SAA1 expression by transcription factor GATAD2A

**Fig. 5:**
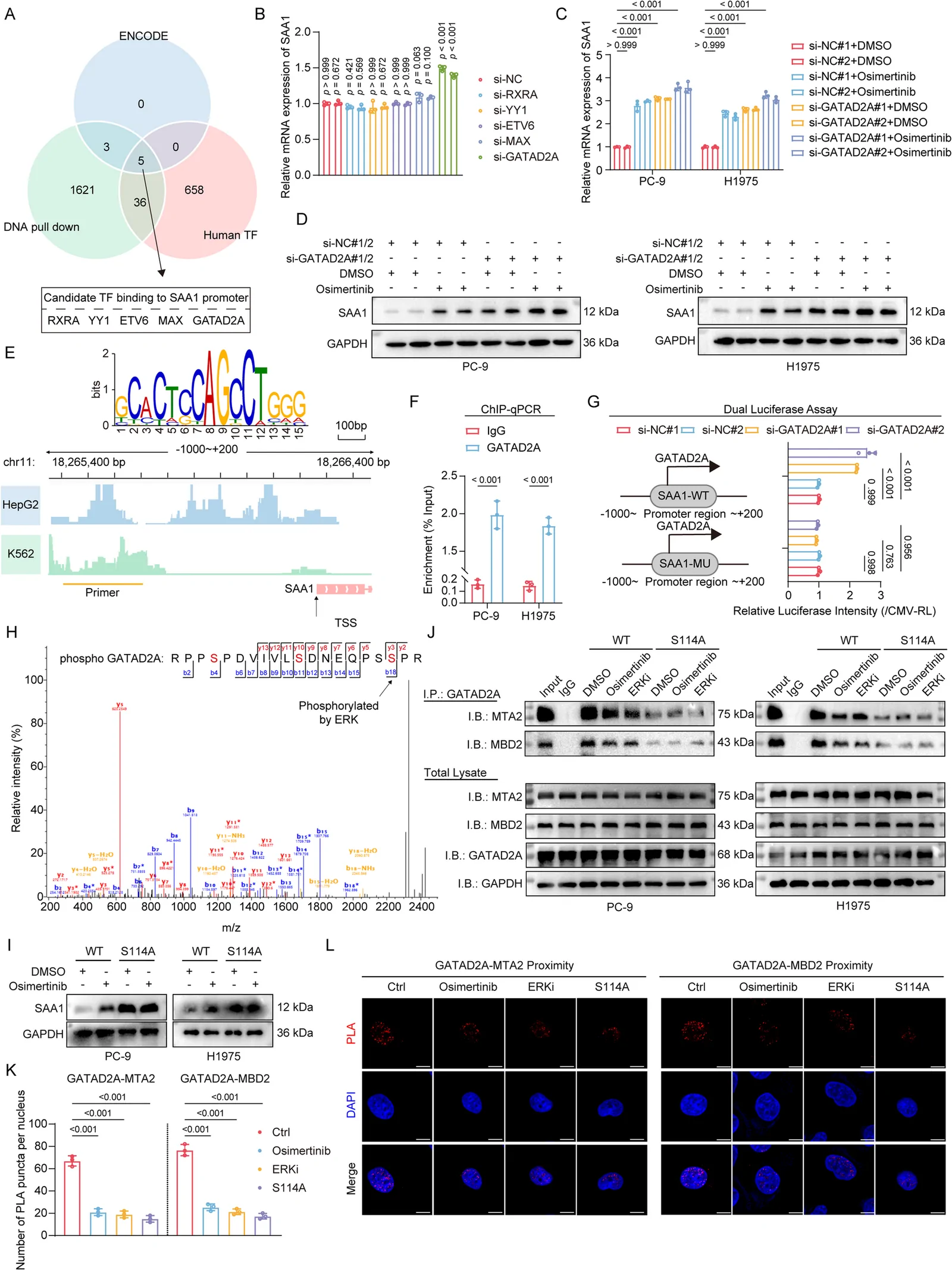
GATAD2A S114 phosphorylation modulates NuRD complex integrity and regulates SAA1 expression. **A** DNA pull-down assay using a biotin-labeled SAA1 promoter probe (-1000 to + 200 bp) incubated with PC-9 nuclear extracts. Mass spectrometry identified 41 transcription factors, five of which (RXRA, YY1, ETV6, MAX, GATAD2A) were selected based on ENCODE ChIP-seq data. **B** qPCR of SAA1 expression following two individual siRNA knockdown of the five candidate transcription factors in PC-9 cells (*n* = 3). **C**, **D** qPCR and western blot of SAA1 levels in PC-9 and H1975 cells transfected with two independent siRNAs targeting GATAD2A (*n* = 3). **E** Predicted GATAD2A binding motif and binding peaks in the SAA1 promoter region from public ChIP-seq datasets, with indicated the location of the primer used for ChIP-qPCR validation. **F** ChIP-qPCR of GATAD2A occupancy at the SAA1 promoter in PC-9 and H1975 cells compared with IgG controls (*n* = 3). **G** Luciferase reporter assay of wild-type and mutant SAA1 promoters in PC-9 and H1975 cells following GATAD2A knockdown (*n* = 3). **H** Phosphoproteomic mapping of GATAD2A in PC-9 parental and OR cells, showing phosphorylated residues including S107, S110 and S114, among which S114 was predicted to be phosphorylated by ERK. I SAA1 expression in PC-9 and H1975 cells harboring the GATAD2A^S114A^ knock-in compared with wild-type cells following osimertinib treatment (10 nM, 48 h). **J** Co-immunoprecipitation analysis of GATAD2A interactions with MBD2 and MTA2 in PC-9 and H1975 cells under different treatments, including osimertinib (10 nM, 48 h), SCH772984 (ERK inhibitor) (10 µM, 48 h), and S114A mutation. **K** Quantitative analysis of the PLA signals representing GATAD2A-NuRD complex interactions in PC-9 and H1975 cells under the indicated conditions (*n* = 3). **L** PLA showing GATAD2A-NuRD component interactions under the same conditions as panel **J**. Dunnett’s multiple comparison tests were applied for panels **B**, **C**, **G**, and **K**; Student’s t-test for panel **F**. Abbreviations: TF, transcription factors; ChIP, chromatin immunoprecipitation; PLA, proximity ligation assay; NuRD, nucleosome remodeling and deacetylase complex

Gene expression can be regulated at multiple levels, including transcriptional control, translational regulation, and post-translational modifications [22–24]. Given the concordance between SAA1 mRNA and protein induction, we investigated transcriptional regulation of SAA1. DNA pull-down assays utilizing a biotinylated SAA1 promoter probe identified 41 candidates, which were refined to five potential regulators (RXRA, YY1, ETV6, MAX, and GATAD2A) by integrating GTRD ChIP-seq datasets (Fig. 5A; Table S6). Functional screening via siRNA-mediated knockdown revealed that only GATAD2A depletion resulted in a robust and reproducible upregulation of SAA1 expression, identifying it as a putative transcriptional repressor (Fig. 5B; Figure S6A-B). Validation in two cell lines confirmed that GATAD2A loss mimics and significantly amplifies osimertinib-induced SAA1 expression at both the transcript and protein levels (Fig. 5C, D; Figure S6C, D).

Mechanistically, ChIP-seq motif analysis identified a GATAD2A binding site near the SAA1 promoter, which was validated by ChIP-qPCR enrichment in PC-9 and H1975 cells (Figs. 5E-F). Functionally, GATAD2A knockdown increased wild-type SAA1 promoter activity, whereas mutation of the binding site abolished this derepression (Fig. 5G). These data conclusively demonstrate that GATAD2A functions as a direct transcriptional repressor of *SAA1*.

### Osimertinib reduces ERK-dependent phosphorylation of GATAD2A at S114, disrupting NuRD complex integrity

We next explored how osimertinib impairs GATAD2A function. Given that post-translational modifications are critical regulators of protein activity, and that EGFR-driven hyperactivation of downstream kinases (e.g., ERK and AKT) is tightly linked to aberrant phosphorylation events, we hypothesized that osimertinib might alter the phosphorylation state of GATAD2A, thereby disrupting its repressive function [25–28].

To identify specific regulatory sites, we performed label-free quantitative phosphoproteomics in PC-9 parental and OR cells. This profiling identified serine 114 (S114) as the most significantly hypophosphorylated residue in the resistant context (Fig. 5H). Bioinformatic analysis using Scansite 4 predicted that the S114 motif is a high-stringency substrate for ERK [29]. Western blot analysis confirmed that osimertinib or ERK inhibition (SCH772984) abolished GATAD2A phosphorylation at S114 without affecting total protein levels (Figure S6E).

Although S114 has been previously cataloged in mass spectrometry datasets, its functional role has remained uncharacterized [30, 31]. To define its role, we generated a phosphorylation-deficient GATAD2A^S114A^ knock-in model using CRISPR/Cas9. These mutant cells exhibited constitutive SAA1 upregulation, phenocopying osimertinib treatment (Fig. 5I).

Mechanistically, since GATAD2A scaffolds the NuRD complex, we hypothesized S114 phosphorylation governs stability [32]. Co-IP and proximity ligation assays (PLA) revealed that osimertinib, ERK inhibition, or the S114A mutation disrupted GATAD2A interaction with core NuRD subunits (MBD2/MTA2) (Figs. 5J-L). Thus, osimertinib-mediated ERK inactivation leads to GATAD2A dephosphorylation, NuRD complex disassembly, and subsequent SAA1 derepression.

### Anti-SAA1 neutralization synergistically enhances osimertinib sensitivity in EGFR-mutant preclinical models

**Fig. 6:**
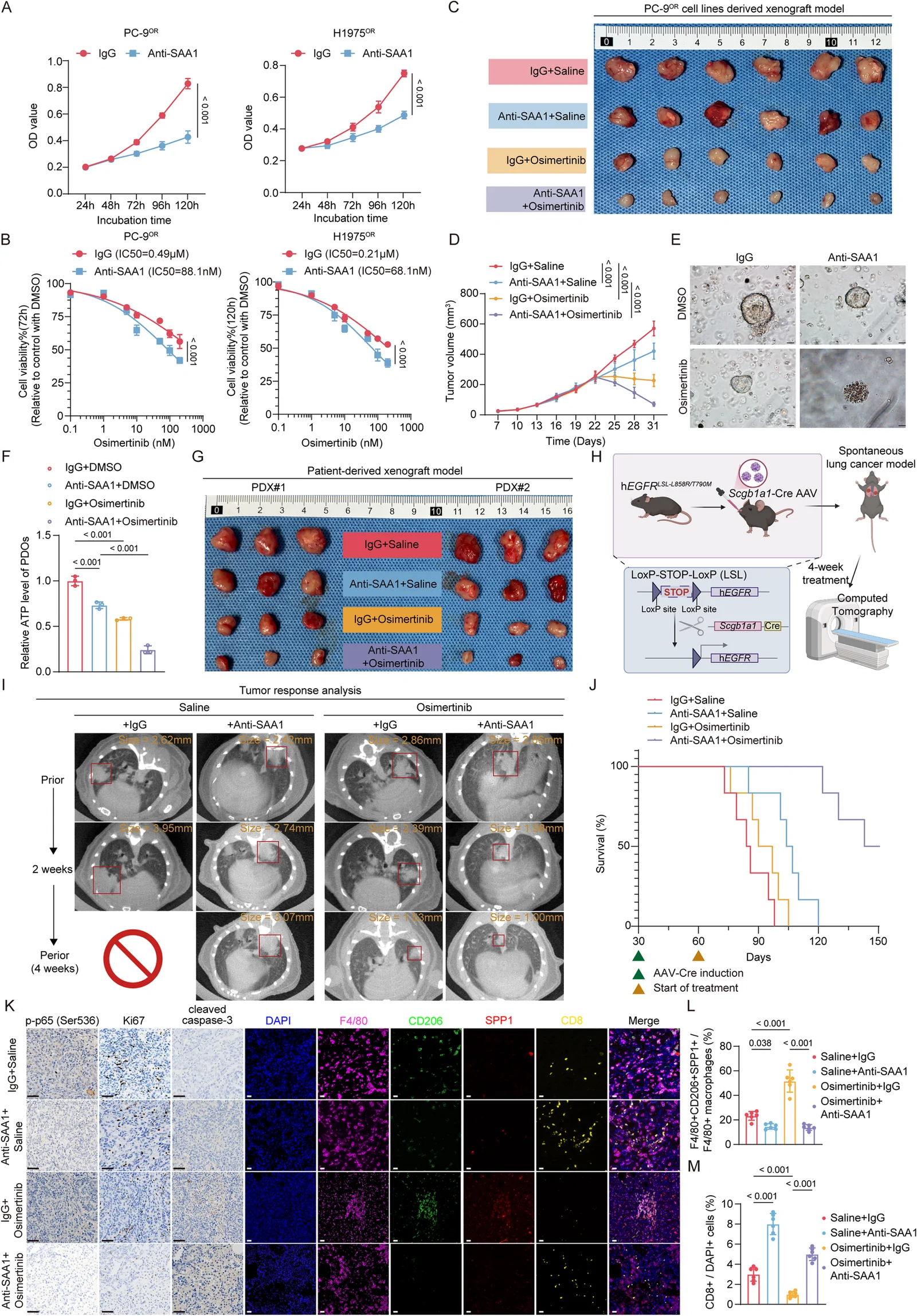
Preclinical evaluation of Anti-SAA1 neutralization in EGFR-mutant models. **A** Proliferation curves of PC-9^OR^ and H1975^OR^ cells treated with Anti-SAA1 (1 µg/mL) or isotype IgG control across the indicated time points (*n* = 3). **B** Relative cell viability of PC-9^OR^ and H1975^OR^ cells following Anti-SAA1 (1 µg/mL) or IgG treatment (*n* = 3). **C**-**D** Representative tumor images (**C**) and tumor growth curves (**D**) of PC-9^OR^-derived CDX models treated with control therapy, Anti-SAA1, osimertinib, or the combination (*n* = 6). Mice were administered daily oral gavage of osimertinib (5 mg/kg) or saline for nine consecutive days. In parallel, intraperitoneal injections of Anti-SAA1 (0.25 µg/g) or control IgG were given every three days (*n* = 6). **E** Morphological features of PDOs treated with IgG, Anti-SAA1 (1 µg/mL) combined with either saline or osimertinib (10 nM). Scale bar, 20 μm. **F** ATP levels in corresponding PDOs under the indicated treatment conditions (*n* = 3). **G** Representative tumor images of PDX models treated with control therapy, Anti-SAA1, osimertinib, or the combination (*n* = 3 for each PDX). **H** Schematic of tumor induction in the h*EGFR*^*L858R/T790M*^ genetically engineered mouse model using intratracheal AAV-*Scgb1a1*-Cre delivery. **I** Micro-CT-based quantification of tumor burden after 4 weeks since treatment with osimertinib alone, Anti-SAA1 plus osimertinib, or isotype control. 30-days after AAV delivery, mice were administered osimertinib (5 mg/kg) or saline by daily oral gavage for 21 consecutive days. In addition, intraperitoneal injections of Anti-SAA1 (0.25 µg/g) or control IgG were given every three days, for a total of six doses. **J** Survival curves of mice receiving the indicated treatments (*n* = 6). **K** Representative immunohistochemical and multiplex immunofluorescence images of lung tumor sections derived from immunocompetent GEMM models following the indicated treatments. DAPI (blue), F4/80 (magenta), CD206 (green), SPP1 (red), and CD8 (yellow). Scale bars, 50 μm. **L**-**M** Quantitative analyses of the percentage of immunosuppressive SPP1^+^CD206^+^ subpopulations among total F4/80^+^ macrophages (**L**) and the infiltration rate of CD8^+^ T cells among total DAPI^+^ cells (**M**) in the spontaneous tumor tissues (*n* = 6). Two-way ANOVA for panels **A**, **B**, and **D**; Dunnett’s multiple comparison test was applied for panel **F**, log-rank test for panel **J**, and one-way ANOVA for panels **L** and **M**. Abbreviations: Anti-SAA1, SAA1 neutralizing antibody; OR, osimertinib-resistant; CDX, cell-derived xenograft; PDO, patient-derived organoid; PDX, patient-derived xenograft; AAV, adeno-associated virus; micro-CT, micro-computed tomography

To assess the translational potential of targeting the SAA1 axis, we employed a neutralizing antibody (Anti-SAA1) validated for cross-reactivity against both human and mouse SAA1 [33]. Dose-titration assays in PC-9^OR^ and H1975^OR^ cells identified 1 µg/mL as the optimal saturating concentration, beyond which no additional inhibitory effect was observed (Figure S7A). At this concentration, Anti-SAA1 markedly suppressed cell proliferation, colony formation, and osimertinib resistance in OR cell lines (Fig. 6A; Figure S7B, C). Crucially, systemic Anti-SAA1 (0.25 µg/g) in C57BL/6 mice elicited no toxicity, with histopathology of major organs confirming a favorable safety profile (Figure S7D). To clarify whether this dependency is strictly restricted to the resistant state, we compared Anti-SAA1 monotherapy across parental and OR models. While SAA1 neutralization exerted a moderate suppressive effect on parental cells, it triggered a drastically more profound growth blockade in OR cells both in vitro (Figure S7E, F) and in CDX models (Figure S7G, H). This parallel comparison confirms that while SAA1 provides a baseline survival advantage, osimertinib-driven resistant cells become uniquely addicted to this induced survival axis.

We next evaluated therapeutic efficacy across multiple in vivo platforms. In PC-9^OR^ CDX model, while monotherapy yielded partial inhibition, the combination of Anti-SAA1 and osimertinib induced overt tumor regression, reducing mean tumor volume to ~ 70 mm^3^ compared to ~ 220 mm^3^ in the osimertinib-only group (Fig. 6C, D). To functionally dissect whether this in vivo efficacy relies on autocrine tumor inhibition or macrophage modulation, we performed macrophage depletion using clodronate liposomes (Figure S7I). Notably, macrophage depletion significantly attenuated the combinatorial efficacy of Anti-SAA1 and osimertinib (Figure S7J), proving that the full therapeutic benefit requires the presence of macrophages, even in an immunodeficient setting. This efficacy was strictly recapitulated in PDOs and PDX. In PDO models, combination treatment disrupted organoid architecture and significantly reduced ATP-based viability. Similarly, in PDX models derived from resistant patients, the combination regimen achieved superior tumor growth control compared to either single agent (Fig. 6E-G; Figure S7K).

Given the dual impact of SAA1 on both tumor cells and macrophages, we further utilized an immunocompetent genetically engineered mouse model (GEMM) harboring the *EGFR*^*L858R/T790M*^ mutations (h*EGFR*^*L858R/T790M*^). Lung tumors were induced by intratracheal delivery of AAV encoding lung-epithelial-specific Cre recombinase (*Scgb1a1*-Cre) in 8-week-old mice (Fig. 6H). Following a 30-day latency for tumor development, mice were randomized to treatment arms. Longitudinal micro-CT imaging revealed that the combination of osimertinib and Anti-SAA1 markedly suppressed tumor burden compared to monotherapy after 4-week evaluation from the start of treatment (Fig. 6I). Most significantly, this regimen conferred a robust survival advantage, extending the median survival from 105.5 days in the osimertinib-treated group to 149.5 days in the combination group (log-rank *p* < 0.001) (Fig. 6J). These results demonstrate that SAA1 neutralization overcomes resistance in both immunodeficient and immunocompetent settings, underscoring its translational relevance.

To elucidate whether this in vivo efficacy is driven by tumor-intrinsic inhibition or microenvironmental remodeling, we profiled the GEMM tumor tissues. Immunohistochemistry confirmed that anti-SAA1 abrogated tumor-intrinsic NF-κB signaling, which translated to robust proliferative arrest and massive apoptosis (Fig. 6K). Concurrently, multiplex immunofluorescence revealed profound immune reprogramming. While osimertinib monotherapy enriched the immunosuppressive SPP1^+^CD206^+^ M2 macrophage population, SAA1 neutralization significantly reduced this subpopulation and increased CD8^+^ T cell infiltration (Fig. 6K-L). Collectively, SAA1 neutralization overcomes resistance through a dual mechanism: promoting tumor cell apoptosis and reversing M2-mediated immunosuppression.

## Discussion

Acquired resistance remains the overarching challenge in the clinical management of EGFR-mutant LUAD, fundamentally limiting the long-term benefit of third-generation TKIs like osimertinib [7, 9, 34]. While late-stage resistance is often attributed to genomic alterations, the early non-genetic adaptations that permit tumor cell survival under drug pressure are poorly defined. Here, we identify a mechanism of therapy-induced secretome remodeling that links initial drug tolerance to stable resistance. We show that SAA1 is not merely an inflammatory bystander but a central orchestrator of the resistant state, coordinating tumor-intrinsic survival signaling with tumor microenvironment reprogramming.

A key conceptual advance of our work is the positioning of SAA1 as an evolutionary bridge connecting initial drug tolerance to stable resistance. SAA1 is classically characterized as an acute-phase reactant synthesized by the liver during systemic inflammation [35]. However, we demonstrate that in the context of TKI therapy, it acts as an autocrine “SOS signal”. This signal is transduced via the TLR4/NF-κB axis. The NF-κB pathway is a master regulator of anti-apoptotic signaling and has been widely implicated in resistance to chemotherapy and targeted agents [36–38]. By activating this pathway, SAA1 elevates the apoptotic threshold, allowing DTPC to survive the initial therapeutic bottleneck. This finding mirrors observations in head and neck squamous cell carcinoma, where NF-κB activation blunts the efficacy of EGFR inhibitors [39]. Importantly, this mechanism operates independently of genomic change, highlighting how adaptive transcriptional programs sustain early survival under kinase inhibition.

Beyond intrinsic survival, SAA1 fundamentally reshapes the TME. Recent studies reveal that EGFR inhibition triggers extensive surfaceome remodeling, exposing new therapeutic vulnerabilities [40]. Complementing these findings, our work highlights that the secreted signaling network is equally dynamic and critical for orchestrating immune evasion. We identify SPP1⁺ macrophages as the dominant effector subset induced by SAA1. Unlike classical M2 macrophages, SPP1⁺ TAMs represent a specialized, pro-angiogenic, and fibrotic population associated with poor immunotherapy outcomes [41, 42]. Importantly, we demonstrate that this SAA1-driven accumulation of SPP1⁺ macrophages creates a physical and functional barrier that spatially excludes cytotoxic CD8⁺ T cells. Notably, we highlight a fascinating cell-type-specific receptor compartmentalization: while SAA1 primarily engages TLR4 to drive tumor-intrinsic survival, it orchestrates the recruitment and polarization of macrophages predominantly via a TLR2-dependent paracrine mechanism. Our macrophage depletion studies (in vivo clodronate liposome assays) unequivocally confirm that this paracrine axis is functionally indispensable for resistant phenotype. We also provide evidence that SAA1 recruits and polarizes macrophages via a TLR4-dependent paracrine mechanism, which expands the concept of therapy adaptation from tumor cell autonomy to the coordinated remodeling of the surrounding immune ecosystem [43–45]. This aligns with spatial transcriptomic findings in liver cancer, where SAA1 dictates macrophage recruitment at the invasive front [20]. This finding expands the concept of therapy adaptation from tumor cell autonomy to coordinated remodeling of the surrounding immune ecosystem.

Mechanistically, we uncover a “therapy paradox” linking kinase inhibition to transcriptional derepression: the pharmacological inhibition of EGFR/ERK—intended to kill the tumor—inadvertently disrupts the epigenetic machinery governing cell identity. Notably, the regulatory serine 114 of GATAD2A resides within a predicted intrinsically disordered region, a structural feature frequently associated with protein–protein interactions [46, 47]. Our data indicate that the loss of ERK-dependent phosphorylation at this site coincides with a compromised interaction between GATAD2A and the NuRD complex core subunits. This destabilization relieves transcriptional repression at the *SAA1* promoter, unleashing its expression [48]. This mechanism illustrates how targeted therapies can inadvertently induce epigenetic instability, permitting stress-responsive gene programs to emerge as adaptive survival strategies.

The dual role of SAA1 makes it an ideal target for combination therapy. Combining a neutralizing SAA1 antibody with osimertinib induced robust tumor regression across multiple preclinical models, including CDX, PDO, PDX, and, most importantly, an immunocompetent spontaneous lung tumor model [33, 49]. This finding is clinically significant as it confirms efficacy in a native immune microenvironment, a limitation of traditional immunodeficient xenografts. This provides a mechanistic rationale for deploying anti-SAA1 strategies to overcome the immunosuppressive “cold” TME characteristic of EGFR-mutant tumors, offering a promising translational avenue to extend the durability of third-generation EGFR-TKIs.

Our study has several limitations. First, while clinical correlations were established, our analysis relied primarily on baseline resected tumors; longitudinal paired biopsies are needed to definitively track SAA1 dynamics during therapy progression. Second, although we identified the TLR4/NF-κB axis and macrophage polarization as key drivers, the potential involvement of alternative receptors (e.g., FPR1) and the complex spatial heterogeneity of the immune microenvironment warrant further characterization using spatial and single-cell transcriptomics. Finally, the broader applicability of the GATAD2A-NuRD regulatory mechanism to other generations of EGFR-TKIs and different cancer contexts remains to be explored in future studies.

## Conclusions

**Fig. 7:**
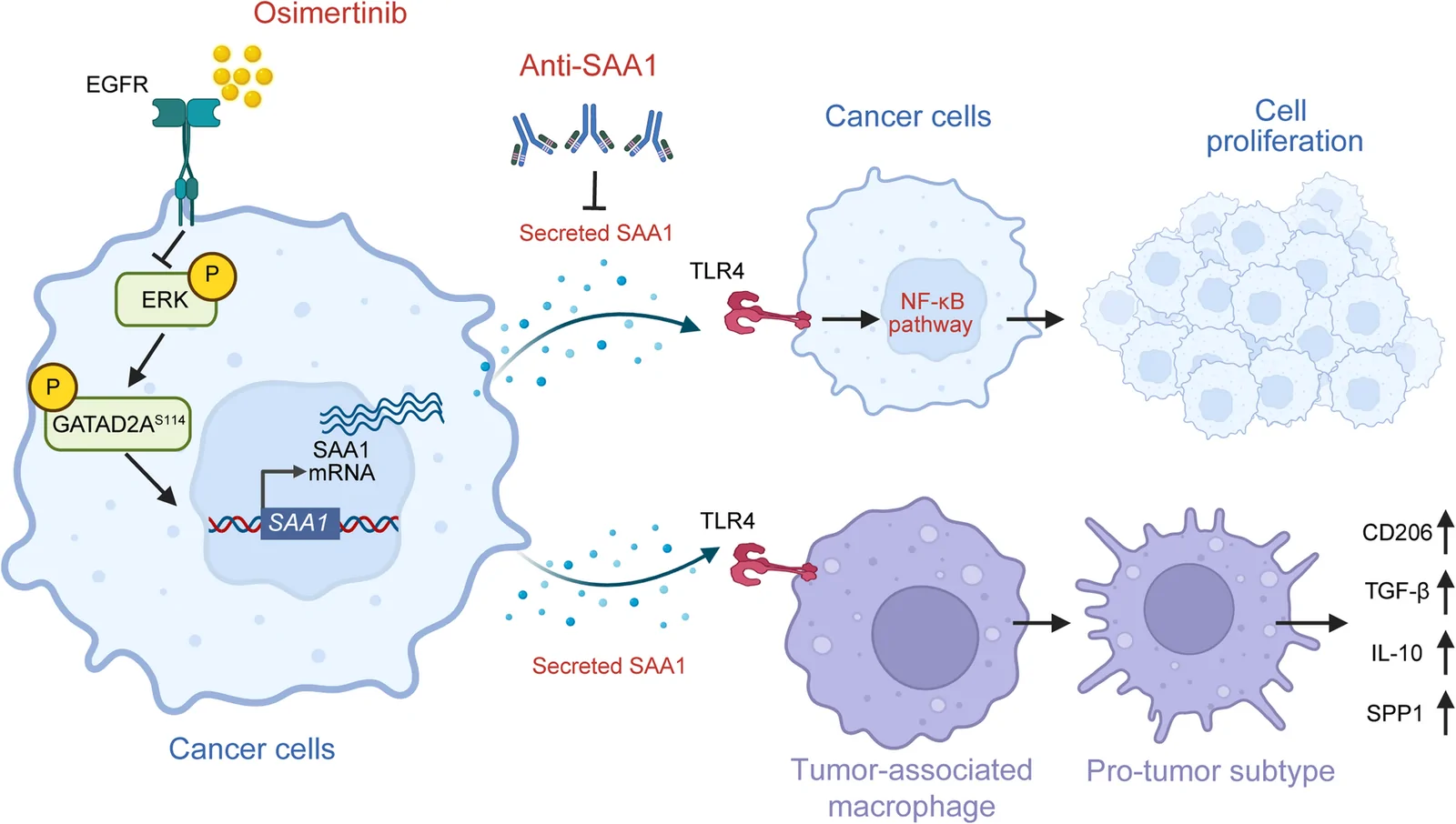
Schematic representation illustrating the mechanism of the ERK/GATAD2A/SAA1 axis in driving osimertinib resistance in EGFR-mutated lung adenocarcinoma. The targeted inhibition of mutant EGFR by osimertinib suppresses downstream ERK signaling. This reduction in ERK kinase activity leads to the dephosphorylation of the epigenetic regulator GATAD2A at the S114 site. In its dephosphorylated state, GATAD2A fails to maintain the assembly of the NuRD corepressor complex at the SAA1 promoter. The dissociation of the NuRD complex relieves the epigenetic silencing of the SAA1 gene, resulting in robust transcriptional derepression and the subsequent secretion of SAA1 protein into the extracellular space. Once secreted, SAA1 functions as a dual-modal mediator to orchestrate acquired resistance. Autocrinely, SAA1 binds to TLR4 on the tumor cell membrane, triggering the intracellular NF-κB signaling cascade to promote tumor cell survival and persistence. Paracrinely, SAA1 remodels the tumor microenvironment by promoting the polarization of macrophages toward an immunosuppressive pro-tumor phenotype. Therapeutic intervention with an anti-SAA1 neutralizing antibody effectively intercepts these extracellular signals, abrogating both the tumor-intrinsic NF-κB survival pathway and the macrophage-mediated immune evasion, thereby restoring sensitivity to osimertinib

Collectively, our findings define an EGFR–GATAD2A–SAA1–TLR4 axis as a central driver of non-genetic resistance. By targeting both the oncogenic driver and the therapy-induced secretome, this dual strategy may extend the durability of third-generation EGFR-TKIs in LUAD (Fig. 7).

### Materials and methods

### Cell lines and cell culture

Human EGFR-mutant LUAD (PC-9, HCC827, H1975), THP-1, and HEK293T cells were obtained from the Chinese National Collection of Authenticated Cell Cultures (Shanghai, China). BMDMs were aseptically isolated from C57BL/6 mice and differentiated with recombinant M-CSF (40 ng/mL; #abs00846, Absin, Shanghai, China). Cells were cultured in DMEM (KeygenBioTECH, Nanjing, China) or RPMI-1640 (KeygenBioTECH) supplemented with 10% fetal bovine serum (FBS; Every Green, Huzhou, China) and penicillin–streptomycin–amphotericin B (Beyotime, Shanghai, China). DTPCs were generated by 10-day high-dose osimertinib treatment, while resistant lines were established via stepwise dose escalation followed by 3-month maintenance (200 nM for PC-9, 1 µM for H1975) [50]. The Transwell inserts used for the co-culture of macrophages and tumor cells were obtained from NEST Biotechnology (Wuxi, China).

### Chemical reagents

Osimertinib (T2490), gefitinib (T1181), afatinib (T21312), the NF-κB inhibitor BAY 11-7082 (T1902), the TLR2 inhibitor C29 (T3502), the TLR4 inhibitor TAK-242 (TQ0181), the ERK inhibitor SCH772984 (T6066), and recombinant human SAA1 (TMPJ-00480) were purchased from TargetMol (Boston, MA, USA). BDTX-1535 (HY-153268) was obtained from MedChemExpress (Monmouth Junction, NJ, USA). All compounds were dissolved in DMSO (Beyotime, Shanghai, China) and stored at − 80 °C.

### Patient cohorts and clinical specimen

Primary LUAD specimens were obtained from surgical resections at Zhongshan Hospital, Fudan University, snap-frozen, and stored at − 80 °C. For paired clinical samples, TKI-naïve specimens were obtained from pre-treatment needle biopsies, and TKI-residual specimens were obtained from surgically resected residual tumors after neoadjuvant osimertinib (Table S1). The percentage of residual viable tumor was assessed by pathological review of the resection specimens. For clinical analysis, 40 EGFR-mutant patients receiving adjuvant osimertinib were retrospectively enrolled and staged according to the 9th edition TNM system (Table S2). Disease-free survival was defined as the interval from surgery to radiologically or pathologically confirmed recurrence or death. The study was approved by the Institutional Review Board of Zhongshan Hospital (Approval No. B2023-258) and conducted with written informed consent in accordance with the Declaration of Helsinki.

### Lentivirus and siRNA

Lentiviral vectors for SAA1 knockout/overexpression and CRISPR/Cas9-mediated GATAD2A^S114A^ knock-in were constructed by GeneChem (Shanghai, China). PC-9 and H1975 cells were infected using HiTrans A (GeneChem) and selected with puromycin (Beyotime). For transient knockdown, siRNAs (GenePharma, Shanghai, China) were transfected at 200 nM using Lipo8000™ (Beyotime). The sequences of all sgRNAs and siRNAs used in this study are provided in Table S3.

### RNA analysis

RNA extraction was conducted following established protocol [51]. Total RNA was extracted from tissues or cells using TRIzol reagent (TIANGEN, Beijing, China). cDNA was synthesized with the Hifair II First-Strand cDNA Synthesis Kit (gDNA Digester Plus; Yeasen, China). Quantitative real-time PCR (qRT–PCR) was performed on an ABI QuantStudio 5 system (Thermo Fisher Scientific) using Hieff^®^ qPCR SYBR Green Master Mix (Low ROX Plus; Yeasen Biotech) and gene-specific primers. Relative gene expression was normalized to β-actin and calculated using the 2^−ΔΔCt method. Primer sequences are listed in Table S4. For RNA sequencing, total RNA was isolated as described above. Library preparation and sequencing were conducted on the Illumina NovaSeq 6000 platform by OE Biotech (Shanghai, China).

### Western blot

Protein extraction and western blot were conducted following established protocol [52]. Lysates (RIPA buffer; Beyotime) were quantified using a BCA Protein Assay Kit (Yeasen Biotechnology), separated by SDS-PAGE (Share-bio, Shang Hai), and transferred to nitrocellulose membranes (Millipore, Germany). Bands were visualized using ECL (New Cell & Molecular Biotech, #P10300) and analyzed via ImageJ. Antibodies included: SAA1 (1:1,000; Proteintech, 16721-1-AP), β-Actin (1:2,500; Beyotime, AF0003), GAPDH (1:2,500; Servicebio, ZB15004-HRP), NF-κB (1:1,000; CST, 8242), p-NF-κB (Ser536) (1:1,000; CST, 3033), IκBα (1:1,000; CST, 4814), p-IκBα (Ser32/36) (1:1,000; CST, 9246), GATAD2A (1:2,000; Proteintech, 12294-1-AP), MTA2 (1:2,000; Proteintech, 17554-1-AP), MBD2 (1:2,000; Proteintech, 55200-1-AP), and p-GATAD2A (Ser114) (1:2,000; ABclonal, custom), TLR2 (1:2,000; Proteintech, 17236-1-AP), TLR4 (1:2,000; Proteintech, 19811-1-AP), anti-histone H3 (1:500; Servicebio, GB11102), RXRA (1:2,000; Proteintech, 21218-1-AP), YY1 (1:2,000; Proteintech, 66281-1-AP), ETV6 (1:2,000; Proteintech, 13182-1-AP), and MAX (1:2,000; Proteintech, 10426-1-AP).

### Enzyme‑linked immunosorbent assay (ELISA)

Cell culture supernatants were collected post-treatment, clarified by centrifugation (300 × g, 10 min), and stored at − 80 °C. Secreted SAA1 levels were quantified using an ELISA kit (EK1280; MultiSciences, Hangzhou, China). Samples and serially diluted standards were incubated in triplicate with biotinylated detection antibodies for 2 h, followed by streptavidin-HRP labeling for 45 min. After TMB substrate development and reaction termination, absorbance was measured at 450 nm, and concentrations were interpolated from the standard curve.

### Cell function assay

Cell proliferation and drug sensitivity were assessed using the CCK-8 assay (TargetMol). For proliferation, 1,500 cells/well were seeded in 96-well plates and monitored over 24–120 h. For drug response, cells (1,500–2,000/well) were treated with EGFR-TKIs for 72 h (PC-9) or 120 h (H1975) prior to absorbance measurement.

For colony formation, 1,000 cells were cultured in 6-well plates for 14 days with medium refreshed every 3 days, followed by fixation (4% formaldehyde), staining (0.5% crystal violet), and quantification via ImageJ. DNA synthesis was analyzed using the EdU Cell Proliferation Kit (E607204, Sangon Biotech) [52]. Treated cells were pulsed with 10 µM EdU for 1–2 h, fixed, permeabilized, and counterstained with Hoechst 33,342 for fluorescence imaging.

To quantify cellular apoptosis following designated treatments, an Annexin V-FITC/PI Apoptosis Detection Kit (KeygenBioTECH) was employed in strict accordance with the manufacturer’s instructions. Briefly, cells were seeded at a density of 1 × 10⁵ cells per well in 12-well culture plates. Following the specific experimental interventions, the cells were gently harvested utilizing trypsinization to ensure the preservation of cell membrane phosphatidylserine integrity. The collected cell populations were then carefully washed, resuspended in the provided binding buffer, and dually stained with Annexin V-FITC and propidium iodide (PI) for 15 min at room temperature, protected from light. The quantification of apoptotic cell fractions was subsequently executed via flow cytometry utilizing a FACScan flow cytometer (Becton Dickinson). The acquired cytometric data, encompassing both early and late apoptotic populations, were comprehensively analyzed and visualized using FlowJo software.

### Macrophage stimulation

THP-1 monocytes were differentiated into M0 macrophages using 100 nM PMA (Sigma-Aldrich) for 48 h, followed by 24 h recovery. BMDMs were differentiated via 7-day M-CSF treatment as described above. For M2 polarization, M0 macrophages were incubated for 48 h with 20 ng/mL IL-4 and IL-13 (human or mouse; Sigma-Aldrich), either alone or combined with TLR4 inhibitors or recombinant SAA1.

### Flow cytometry

Flow cytometry was conducted to detect the polarization of macrophages based on former study [53]. Single-cell suspensions were prepared by digestion and filtration (40 μm). Dead cells were excluded using Fixable Viability Dye eFluor (65-0866-14, Thermo Fisher). Following Fc blocking (CD16/CD32; Thermo Fisher), cells were stained with anti-CD86-APC (#305412) and anti-CD206-PE (#321106) from BioLegend. Data were acquired on a BD FACSAria III and analyzed using FlowJo v10.

### Multiplex immunohistochemistry (mIHC) and immunofluorescence staining

Paraffin-embedded Sect.  (5 μm) underwent deparaffinization, rehydration, and citrate buffer antigen retrieval (pH 8.0; Beyotime). For IHC, slides were blocked and incubated overnight with anti-SAA1 (1:200; Proteintech, 16721-1-AP), anti-Ki67 (1:500; Servicebio, GB1211441), anti-cleaved caspase-3 (1:500; Servicebio, GB11532), and p-NF-κB (Ser536) (1:200; CST, 3033). Detection was performed using HRP-conjugated secondary antibodies (1:500; Vector Laboratories) and DAB substrate (Dako), followed by hematoxylin counterstaining. Images were acquired on Nikon Eclipse E100 or Olympus BX51 microscopes. IHC staining was semi-quantitatively evaluated using an H-score system. The H-score was calculated as follows: H-score = 1 × percentage of weakly stained tumor cells + 2 × percentage of moderately stained tumor cells + 3 × percentage of strongly stained tumor cells, with a possible range of 0–300. Based on the calculated H-scores, the overall staining was stratified into four categories: poor/weak (0–75), mild (75–150), moderate (150–225), and strong (225–300). For survival analysis, tumors with moderate or strong baseline SAA1 staining were classified as the SAA1-high group, whereas tumors with poor/weak or mild staining were classified as the SAA1-low group.

Multiplex immunofluorescence was performed by Servicebio (Wuhan, China) using sequential Tyramide Signal Amplification (TSA). Cycles involved primary antibody incubation, HRP-secondary labeling, TSA amplification (Servicebio), and microwave-mediated antibody stripping. Antibodies included anti-F4/80 (GB113373), anti-CD206 (GB113497), anti-CD68 (GB113150), anti-SPP1 (GB120020), anti-CD8 alpha (GB15068) (all 1:500; Servicebio). Signals were visualized using iF488/546/647/700-TSA (Servicebio) with DAPI counterstaining.

### Chromatin immunoprecipitation (ChIP) assay

Enzymatic Chromatin IP Kit (Magnetic Beads) (Cell Signaling Technology, CST) following the manufacturer’s recommendations and established laboratory procedures [50]. Cross-linked chromatin was digested with micrococcal nuclease and incubated with anti-GATAD2A (Proteintech, 12294-1-AP), anti-Histone H3 (positive control, CST), or rabbit IgG (negative control, CST). DNA-protein complexes captured on Protein G magnetic beads were washed, eluted, and purified for qPCR analysis using primers listed in Table S4.

### Co-immunoprecipitation (Co-IP) assay

For Co-IP, cells were lysed in NP-40 buffer (Beyotime) with protease/phosphatase inhibitors (TargetMol). Lysates were incubated with Protein A/G magnetic beads (Thermo Fisher Scientific) pre-conjugated to antibodies via disuccinimidyl suberate (DSS) crosslinking. Complexes were eluted for Western blot or mass spectrometry.

### Dual-luciferase reporter assay

Wild-type or mutant SAA1 promoter constructs cloned into pGL4 vectors (Genechem) were co-transfected into HEK293T cells with a Renilla luciferase control using Lipo8000 (Beyotime). For mechanistic validation, cells were co-transfected with GATAD2A-targeting siRNAs. Activity was quantified at 48 h using the Dual Luciferase Reporter Gene Assay Kit (Beyotime).

### DNA affinity precipitation assay

DNA affinity precipitation assays were conducted following previously reported procedures [54, 55]. Biotinylated SAA1 promoter probes (− 1000 to + 200 bp) were amplified (primers: Table S4; Sangon) and purified using the MiniBEST Kit (Takara). Nuclear extracts (200 µg; Beyotime kit with TargetMol inhibitors) were incubated with 1 µg probe-conjugated streptavidin beads (CST) overnight at 4 °C. Nonspecific binding was suppressed using salmon sperm DNA (Solarbio) and BSA (Sangon), while specificity was confirmed with 20-fold excess unlabeled competitor. Eluted complexes were analyzed by Western blot or LC-MS/MS using an Easy-nLC 1200/Q Exactive system (Thermo Fisher). Proteomic data were processed via Proteome Discoverer 2.5 (results in Table S5).

### Phosphoproteomics analysis

Phosphoproteomic analysis was performed by Shanghai Luming Biological Technology using a 4D label-free workflow. Tryptic peptides (Beyotime) were enriched via IMAC (Thermo Fisher Scientific) and analyzed on an EASY-nLC 1200 coupled to a timsTOF Pro 2. Data were processed using MaxQuant (v1.6.17.0) against the UniProt human reference proteome (Feb 2023) [56]. Phosphorylation site identification, quantification, and downstream analyses were conducted following the same criteria and parameters as previously reported [57].

### Proximity ligation assay (PLA)

PLA was conducted using the Duolink In Situ PLA kit (Sigma–Aldrich) according to our previously published protocol [58]. Cells were fixed (4% PFA), permeabilized (0.1% Triton X-100), and blocked (3% BSA). Interactions were probed overnight with anti-GATAD2A (1:200; Proteintech, 12294-1-AP) paired with anti-MBD2 (1:200; Santa Cruz, sc-514062) or anti-MTA2 (1:200; Proteintech, 66195-1-Ig). Detection was performed using Duolink PLA probes (anti-rabbit MINUS/anti-mouse PLUS) with ligation and amplification per manufacturer. Fluorescent puncta were visualized using a Nikon ECLIPSE TI confocal microscope with DAPI counterstaining.

### Cell-derived xenograft (CDX) model

CDX models were established by subcutaneously injecting 1 × 10^6^ PC-9^OR^ cells (control, SAA1-targeting, or parental) into 4-week-old male BALB/c nude mice. Tumor volumes were calculated as (length × width²) / 2. Mice received oral osimertinib (5 mg/kg, daily) or intraperitoneal anti-SAA1 neutralizing antibody (0.25 µg/g, every 3 days; R&D Systems, #AF2948) versus isotype IgG (Beyotime) [33]. For in vivo macrophage depletion experiments, macrophage depletion was initiated on day 18 post-tumor engraftment via injections of clodronate liposomes (Yeasen Biotechnology, #40337ES10) or control liposomes (Yeasen Biotechnology, #40338ES) (200 µL), followed by maintenance injections (200 µL) every three days. The anti-SAA1 antibody cross-reacts with human and mouse SAA1. All animal procedures were conducted in accordance with institutional guidelines and were approved by the relevant animal ethics committee.

### Patient-derived xenograft (PDX) model

PDX models were established using fresh EGFR-mutant LUAD specimens obtained from Zhongshan Hospital, Fudan University. Tumor fragments (2–3 mm³) were subcutaneously implanted into 4-week-old male NSG mice and serially passaged (P1–P2) to generate experimental cohorts. Upon tumor establishment, mice were randomized to receive vehicle, osimertinib alone, Anti-SAA1 alone, or combination therapy. Osimertinib was administered via oral gavage (5 mg/kg, daily), while Anti-SAA1 (R&D Systems) or isotype IgG (Beyotime) was injected intraperitoneally (0.25 µg/g, every 3 days). All procedures were approved by the Zhongshan Hospital IACUC.

### Patient-derived organoids (PDO) and PDO viability assays

PDOs were established from EGFR-mutant LUAD tissues via enzymatic digestion and embedding in growth factor–reduced Matrigel (D1 Medical Technology) with the human lung cancer PDO culture medium kit (Absin, Shanghai, China). Organoids were treated with osimertinib (10 nM) and/or Anti-SAA1 (15 µg/mL; R&D Systems) versus IgG control (Beyotime). Growth and viability were assessed by microscopy and ATP quantification using the CellTiter-Lumi 3D Cell Viability Assay (Beyotime).

### Spontaneous lung cancer model

Spontaneous lung adenocarcinoma was induced in 8-week-old h*EGFR*^*L858R/T790M*^ mice via intratracheal administration of AAV-*Scgb1a1*-Cre to activate mutant EGFR expression in airway epithelium. Following 30 days of tumor development, mice were randomized to receive vehicle, osimertinib monotherapy, or combination therapy. Osimertinib was administered via oral gavage (5 mg/kg, daily), while Anti-SAA1 neutralizing antibody (R&D Systems) or isotype IgG control (Beyotime) was delivered via intraperitoneal injection (0.25 µg/g, every 3 days). Tumor burden was monitored longitudinally using micro-computed tomography (micro-CT), and all procedures were approved by the Ethics Committee of Zhongshan Hospital, Fudan University.

### Data processing and statistics

LUAD transcriptomic and clinical data were obtained from TCGA. TF binding sites were predicted using ENCODE and MEME Suite [59, 60]. Functional enrichment (GO, KEGG, GSEA) was performed using the R package clusterProfiler, and differential expression was analyzed via limma [61, 62]. For secreted proteins, a threshold of |log_2_FC| > 0.75 was applied (osimertinib vs. DMSO). Candidate TFs were selected based on enrichment in biotin-pull-down assays and documented SAA1 promoter binding in the GTRD [63]. Statistical analyses were conducted using GraphPad Prism 10 (*p* < 0.05 considered significant). All experiments were performed in triplicate unless otherwise stated.

## Supplementary Information

Supplementary Material 1.

Supplementary Material 2: Supplementary Figure 1 (referring to Figure 1) A-B Relative cell viability of PC-9^OR^ (A) and H1975^OR^ (B) cells treated with gefitinib, afatinib, or the fourth-generation EGFR inhibitor BDTX-1535 across the indicated concentration ranges (*n* = 3). C-D Cell proliferation assays performed in PC-9^OR^ (C) and H1975^OR^ (D) cells under treatment with gefitinib, afatinib, or BDTX-1535 for the indicated durations (*n* = 3). E qPCR and western blot quantification of SAA1 expression in paired TKI-naïve and TKI-residual EGFR-mutant LUAD samples collected before and after neoadjuvant osimertinib treatment (*n* = 5). EGFR-TKI were used at a dose of 10 nM. Two-way ANOVA tests were applied for panel A-D, Paired Student’s t-test for panel E. Abbreviations: OR, osimertinib-resistant; Osi, osimertinib; Gef, gefitinib; Afa, afatinib. Supplementary Figure 2 (referring to Figure 2) A-B qPCR and western blot assessing SAA1 knockdown efficiency in PC-9 and H1975 cells transfected with two independent siRNAs targeting SAA1 (*n* = 3). C-D Colony formation assays performed in PC-9^OR^ and H1975^OR^ cells expressing vector control, SAA1-OE, or SAA1-KO constructs, in the presence of DMSO or osimertinib (200 nM) (*n* = 3). E EdU incorporation assays under the same experimental conditions as in panels C-D to evaluate proliferation in PC-9^OR^ and H1975^OR^ cells with vector, SAA1-OE, or SAA1-KO backgrounds (*n* = 3). Scale bar, 20 μm. F-G Representative flow cytometry plots (F) and quantitative analysis (G) of Annexin V/PI apoptosis assays in PC-9^OR^ and H1975^OR^ cells expressing vector, SAA1-OE, SAA1-KO, or SAA1-KO rescued with exogenous SAA1 (100 ng/ml), treated with DMSO or osimertinib (200 nM) (*n* = 3). H DTPC emergence and survival curves monitoring cell counts over 60 days of continuous osimertinib treatment in PC-9 and H1975 cells with vector control, SAA1-OE, or SAA1-KO, evaluating the delay in acquired resistance. I Cell proliferation curves of parental and osimertinib-resistant PC-9 and H1975 cells stably expressing control vector or SAA1-KO constructs (*n* = 3). J Relative inhibition rates of parental and osimertinib-resistant cells treated with osimertinib, normalized to their respective vector-transfected controls (*n* = 3). K Representative IHC staining of SAA1, the proliferation marker Ki67, and the apoptosis marker cleaved caspase-3 in tumor sections derived from CDX models treated with saline or osimertinib, comparing vector control and SAA1-KO groups. Scale bar, 25 μm. One-way ANOVA or two-way ANOVA tests with Dunnett’s multiple comparison tests were applied for panels A, C, D, G, and J; Student’s t-test was applied for panel I. Abbreviations: SAA1-OE, SAA1 overexpression; SAA1-KO, SAA1 knockout; OR, osimertinib-resistant; PR, parental; DTPC, drug-tolerant persister cells; CDX, cell-derived xenograft. Supplementary Figure 3 (referring to Figure 3) A GO and KEGG pathway enrichment analysis of TCGA-LUAD samples stratified into SAA1-low and SAA1-high groups. B Heatmap displaying expression patterns of NF-κB-related genes in TCGA-LUAD SAA1-low versus SAA1-high groups. C-D KEGG pathway enrichment analysis of PC-9 (C) and H1975 (D) cells treated with PBS or exogenous SAA1 (100 ng/ml, 24 h). E Heatmap showing NF-κB-related gene expression in H1975 cells treated with PBS or exogenous SAA1. F-G Western blot of NF-κB pathway–associated proteins in whole-cell lysates (F) and subcellular fractions (G) across PR, DTPC, and OR cell models. H-I Relative mRNA expression of classical downstream NF-κB target genes (*NFKBIA*, *BCL2*, *CXCL8*, *IL6*) evaluated by qPCR in PR, DTPC, and OR models (H), and in OR models with genetic manipulation of SAA1 (I) (*n* = 3). Dunnett’s multiple comparison tests were applied for panels H-I. Abbreviations: LUAD, lung adenocarcinoma; PR, parental; DTPC, drug-tolerant persister cells; OR, osimertinib-resistant. Supplementary Figure 4 (referring to Figure 3) A-B Validation of siRNA-mediated knockdown efficiency against TLR2 and TLR4 at the mRNA (A) and protein (B) levels in PC-9^OR^ and H1975^OR^ cells using two independent siRNAs (*n* = 3). C Relative cell viability assays comparing the sensitizing effects of TLR2 versus TLR4 knockdown on osimertinib treatment (n = 3). D-E Western blot analysis of whole-cell lysates (D) and subcellular fractions (E) demonstrating that specific TLR4 depletion abolishes exogenous SAA1-induced p-p65 activation and its nuclear translocation. F-G Relative mRNA expression of downstream NF-κB targets (*NFKBIA*, *BCL2*, *CXCL8*, *IL6*) in PC-9^OR^ (F) and H1975^OR^ (G) cells (*n* = 3). H-I Representative flow cytometry plots (H) and quantitative bar charts (I) of Annexin V/PI apoptosis assays in OR cells treated with DMSO or osimertinib, assessing the anti-apoptotic effect of exogenous SAA1 and its reversal by co-treatment with NF-κBi or TLR4i (*n* = 3). Dunnett’s multiple comparison tests were applied for panels A, F, G, and I; Two-way ANOVA test was applied for panel C. Abbreviations: si-NC, negative control siRNA; OR, osimertinib-resistant; NF-κBi, NF-κB inhibitor; TLR4i, TLR4 inhibitor. Supplementary Figure 5 (referring to Figure 4) A qPCR analysis of M2 marker expression in macrophages treated with recombinant SAA1 protein compared with untreated controls (*n* = 3). B qPCR analysis of classical M2-associated markers in H1975 osimertinib-resistant cells co-cultured with macrophages under the indicated conditions (*n* = 3). C qPCR analysis of M2 markers in macrophages treated with exogenous SAA1 in the presence or absence of C29 (50μM) (n = 3). D-E Flow cytometry plots (E) and corresponding quantification (D) evaluating the proportion of CD206⁺ M2 macrophages in BMDM cells treated with PBS or exogenous SAA1 (100 ng/ml, 24h), combined with DMSO, TAK-242 (10μM, 24h), or C29 (50μM, 24h) (*n* = 3). Student’s t-test was for panel B and one-way or two-way ANOVA with Dunnett’s multiple comparison tests were for panels A, C, D. Supplementary Figure 6 (referring to Figure 5) A qPCR analysis and western blots showing knockdown efficiency for each transcription factor using the corresponding siRNAs. C-D qPCR and western blot validation of GATAD2A knockdown efficiency using two independent siRNAs (*n* = 3). E Western blot analysis of GATAD2A and p-GATAD2A (S114) levels in cells treated with osimertinib (10 nM, 48 h) or an SCH772984 (ERK inhibitor) (10 μM, 48 h). Dunnett’s multiple comparison was applied for panels A and B. Supplementary Figure 7 (referring to Figure 6) A Dose-response analysis of Anti-SAA1 in PC-9^OR^ and H1975^OR^ cells (n = 3). B-C Effects of Anti-SAA1 (1 μg/mL) on colony formation and EdU incorporation experiments PC-9^OR^ and H1975^OR^ cells (n = 3). Scale bar, 20 μm. D H&E staining of major organs (heart, liver, kidney, spleen, and lung) from C57BL/6 mice systemically treated with Anti-SAA1 (0.25 μg/g). E Cell proliferation assays comparing the effect of Anti-SAA1 (1 μg/mL) versus isotype IgG monotherapy on parental and osimertinib-resistant PC-9 and H1975 cell lines over the indicated time points (n = 5). F Relative cell viability of PR and OR cells (PC-9 and H1975) at 120 h post-treatment with Anti-SAA1, normalized to their respective IgG controls (n = 5). G Tumor growth curves of PC-9 PR and OR cell-derived xenograft models treated with Anti-SAA1 (0.25 μg/g) or isotype IgG monotherapy (n = 6). H Relative tumor volume of PR and OR CDX models at the endpoint (Day 31) following Anti-SAA1 monotherapy, normalized to their respective IgG control groups (n = 6). I Representative immunofluorescence images of F4/80 staining for the evaluation of in vivo macrophage depletion efficiency in the CDX models. Tumor sections from the Control liposomes and Clodronate liposomes groups were stained for F4/80 (green) and DAPI (blue). Scale bar, 20 µm. J Tumor growth curves of PC-9^OR^-derived CDX models treated with osimertinib, with or without Anti-SAA1, in macrophage-intact (control liposomes) or macrophage-depleted (clodronate liposomes) mice (n = 6). Mice were administered daily oral gavage of osimertinib (5 mg/kg). In parallel, intraperitoneal injections of Anti-SAA1 (0.25 μg/g) or control IgG were given every three days. Macrophage depletion was initiated on day 18 post-tumor engraftment via injections of clodronate liposomes or control liposomes (200 μL), followed by maintenance injections (200 μL) every three days (n = 6). K Tumor weight of PDX models treated with control therapy, Anti-SAA1, osimertinib, or the combination (n = 3 for each PDX). Scale bar, 50 μm. Dunnett’s multiple comparison tests were applied for panels A and K; Student’s t-test was applied for panels B, F, and H, and two-way ANOVA was applied for panel J. Abbreviations: PR, parental; OR, osimertinib resistant; CDX, cell-derived xenografts; H&E, hematoxylin and eosin; PDX, patient-derived xenografts.

## Data Availability

The data analyzed or used in this study are available from the corresponding author on reasonable request.

## Abbreviations

AAV: Adeno-associated virus
BMDM: Bone marrow-derived macrophage
CDX: Cell-derived xenograft
ChIP: Chromatin immunoprecipitation
Co-IP: Co-immunoprecipitation
DTPC: Drug-tolerant persister cell
EGFR: Epidermal growth factor receptor
ELISA: Enzyme-linked immunosorbent assay
GO: Gene Ontology
GSEA: Gene Set Enrichment Analysis
IC50: Half-maximal inhibitory concentration
IHC: Immunohistochemistry
KEGG: Kyoto Encyclopedia of Genes and Genomes
LUAD: Lung adenocarcinoma
micro-CT: Micro-computed tomography
NuRD: Nucleosome remodeling and deacetylase
OR: Osimertinib-resistant
PDO: Patient-derived organoid
PDX: Patient-derived xenograft
PLA: Proximity ligation assay
SAA1: Serum amyloid A1
TAM: Tumor-associated macrophage
TKI: Tyrosine kinase inhibitor
TME: Tumor microenvironment
