# Supplementary material for: ERK/GATAD2A/SAA1 axis drives osimertinib resistance via NF-κB and macrophage reprogramming in EGFR-mutated lung adenocarcinoma
Source: J Exp Clin Cancer Res. 2026 Jul 28;45:221. doi: 10.1186/s13046-026-03792-6 (PMC13693122; doi:10.1186/s13046-026-03792-6)
Supplement: Supplementary file 1 [file 13046_2026_3792_MOESM2_ESM.docx]

**Supplementary Material**

**Supplementary Fig. 1 (referring to Fig. 1)** A-B Relative cell viability of PC-9^OR^ (A) and H1975^OR^ (B) cells treated with gefitinib, afatinib, or the fourth-generation EGFR inhibitor BDTX-1535 across the indicated concentration ranges (*n* = 3). C-D Cell proliferation assays performed in PC-9^OR^ (C) and H1975^OR^ (D) cells under treatment with gefitinib, afatinib, or BDTX-1535 for the indicated durations (*n* = 3). E qPCR and western blot quantification of SAA1 expression in paired TKI-naïve and TKI-residual EGFR-mutant LUAD samples collected before and after neoadjuvant osimertinib treatment (*n* = 5). EGFR-TKI were used at a dose of 10 nM. Two-way ANOVA tests were applied for panel A-D, Paired Student’s t-test for panel E. Abbreviations: OR, osimertinib-resistant; Osi, osimertinib; Gef, gefitinib; Afa, afatinib.

**Supplementary Fig. 2 (referring to Fig. 2)** A-B qPCR and western blot assessing SAA1 knockdown efficiency in PC-9 and H1975 cells transfected with two independent siRNAs targeting SAA1 (*n* = 3). C-D Colony formation assays performed in PC-9^OR^ and H1975^OR^ cells expressing vector control, SAA1-OE, or SAA1-KO constructs, in the presence of DMSO or osimertinib (200 nM) (*n* = 3). E EdU incorporation assays under the same experimental conditions as in panels C-D to evaluate proliferation in PC-9^OR^ and H1975^OR^ cells with vector, SAA1-OE, or SAA1-KO backgrounds (*n* = 3). Scale bar, 20 μm. F-G Representative flow cytometry plots (F) and quantitative analysis (G) of Annexin V/PI apoptosis assays in PC-9^OR^ and H1975^OR^ cells expressing vector, SAA1-OE, SAA1-KO, or SAA1-KO rescued with exogenous SAA1 (100 ng/ml), treated with DMSO or osimertinib (200 nM) (*n* = 3). H DTPC emergence and survival curves monitoring cell counts over 60 days of continuous osimertinib treatment in PC-9 and H1975 cells with vector control, SAA1-OE, or SAA1-KO, evaluating the delay in acquired resistance. I Cell proliferation curves of parental and osimertinib-resistant PC-9 and H1975 cells stably expressing control vector or SAA1-KO constructs (*n* = 3). J Relative inhibition rates of parental and osimertinib-resistant cells treated with osimertinib, normalized to their respective vector-transfected controls (*n* = 3). K Representative IHC staining of SAA1, the proliferation marker Ki67, and the apoptosis marker cleaved caspase-3 in tumor sections derived from CDX models treated with saline or osimertinib, comparing vector control and SAA1-KO groups. Scale bar, 25 μm. One-way ANOVA or two-way ANOVA tests with Dunnett’s multiple comparison tests were applied for panels A, C, D, G, and J; Student’s t-test was applied for panel I. Abbreviations: SAA1-OE, SAA1 overexpression; SAA1-KO, SAA1 knockout; OR, osimertinib-resistant; PR, parental; DTPC, drug-tolerant persister cells; CDX, cell-derived xenograft.

**Supplementary Fig. 3 (referring to Fig. 3)** A GO and KEGG pathway enrichment analysis of TCGA-LUAD samples stratified into SAA1-low and SAA1-high groups. B Heatmap displaying expression patterns of NF-κB-related genes in TCGA-LUAD SAA1-low versus SAA1-high groups. C-D KEGG pathway enrichment analysis of PC-9 (C) and H1975 (D) cells treated with PBS or exogenous SAA1 (100 ng/ml, 24 h). E Heatmap showing NF-κB-related gene expression in H1975 cells treated with PBS or exogenous SAA1. F-G Western blot of NF-κB pathway–associated proteins in whole-cell lysates (F) and subcellular fractions (G) across PR, DTPC, and OR cell models. H-I Relative mRNA expression of classical downstream NF-κB target genes (*NFKBIA*, *BCL2*, *CXCL8*, *IL6*) evaluated by qPCR in PR, DTPC, and OR models (H), and in OR models with genetic manipulation of SAA1 (I) (*n* = 3). Dunnett’s multiple comparison tests were applied for panels H-I. Abbreviations: LUAD, lung adenocarcinoma; PR, parental; DTPC, drug-tolerant persister cells; OR, osimertinib-resistant.

**Supplementary Fig. 4 (referring to Fig. 3)** A-B Validation of siRNA-mediated knockdown efficiency against TLR2 and TLR4 at the mRNA (A) and protein (B) levels in PC-9^OR^ and H1975^OR^ cells using two independent siRNAs (*n* = 3). C Relative cell viability assays comparing the sensitizing effects of TLR2 versus TLR4 knockdown on osimertinib treatment (*n* = 3). D-E Western blot analysis of whole-cell lysates (D) and subcellular fractions (E) demonstrating that specific TLR4 depletion abolishes exogenous SAA1-induced p-p65 activation and its nuclear translocation. F-G Relative mRNA expression of downstream NF-κB targets (*NFKBIA*, *BCL2*, *CXCL8*, *IL6*) in PC-9^OR^ (F) and H1975^OR^ (G) cells (*n* = 3). H-I Representative flow cytometry plots (H) and quantitative bar charts (I) of Annexin V/PI apoptosis assays in OR cells treated with DMSO or osimertinib, assessing the anti-apoptotic effect of exogenous SAA1 and its reversal by co-treatment with NF-κBi or TLR4i (*n* = 3). Dunnett’s multiple comparison tests were applied for panels A, F, G, and I; Two-way ANOVA test was applied for panel C. Abbreviations: si-NC, negative control siRNA; OR, osimertinib-resistant; NF-κBi, NF-κB inhibitor; TLR4i, TLR4 inhibitor.

**Supplementary Fig. 5 (referring to Fig. 4)** A qPCR analysis of M2 marker expression in macrophages treated with recombinant SAA1 protein compared with untreated controls (*n* = 3). B qPCR analysis of classical M2-associated markers in H1975 osimertinib-resistant cells co-cultured with macrophages under the indicated conditions (*n* = 3). C qPCR analysis of M2 markers in macrophages treated with exogenous SAA1 in the presence or absence of C29 (50μM) (*n* = 3). D-E Flow cytometry plots (E) and corresponding quantification (D) evaluating the proportion of CD206⁺ M2 macrophages in BMDM cells treated with PBS or exogenous SAA1 (100 ng/ml, 24h), combined with DMSO, TAK-242 (10μM, 24h), or C29 (50μM, 24h) (*n* = 3). Student’s t-test was for panel B and one-way or two-way ANOVA with Dunnett’s multiple comparison tests were for panels A, C, D.

**Supplementary Fig. 6 (referring to Fig. 5)** A qPCR analysis and western blots showing knockdown efficiency for each transcription factor using the corresponding siRNAs. C-D qPCR and western blot validation of GATAD2A knockdown efficiency using two independent siRNAs (*n* = 3). E Western blot analysis of GATAD2A and p-GATAD2A (S114) levels in cells treated with osimertinib (10 nM, 48 h) or an SCH772984 (ERK inhibitor) (10 μM, 48 h). Dunnett’s multiple comparison was applied for panels A and B.

**Supplementary Fig. 7 (referring to Fig. 6)** A Dose-response analysis of Anti-SAA1 in PC-9^OR^ and H1975^OR^ cells (*n* = 3). B-C Effects of Anti-SAA1 (1 μg/mL) on colony formation and EdU incorporation experiments PC-9^OR^ and H1975^OR^ cells (*n* = 3). Scale bar, 20 μm. D H&E staining of major organs (heart, liver, kidney, spleen, and lung) from C57BL/6 mice systemically treated with Anti-SAA1 (0.25 μg/g). E Cell proliferation assays comparing the effect of Anti-SAA1 (1 μg/mL) versus isotype IgG monotherapy on parental and osimertinib-resistant PC-9 and H1975 cell lines over the indicated time points (*n* = 5). F Relative cell viability of PR and OR cells (PC-9 and H1975) at 120 h post-treatment with Anti-SAA1, normalized to their respective IgG controls (n = 5). G Tumor growth curves of PC-9 PR and OR cell-derived xenograft models treated with Anti-SAA1 (0.25 μg/g) or isotype IgG monotherapy (*n* = 6). H Relative tumor volume of PR and OR CDX models at the endpoint (Day 31) following Anti-SAA1 monotherapy, normalized to their respective IgG control groups (n = 6). I Representative immunofluorescence images of F4/80 staining for the evaluation of *in vivo* macrophage depletion efficiency in the CDX models. Tumor sections from the Control liposomes and Clodronate liposomes groups were stained for F4/80 (green) and DAPI (blue). Scale bar, 20 µm. J Tumor growth curves of PC-9^OR^-derived CDX models treated with osimertinib, with or without Anti-SAA1, in macrophage-intact (control liposomes) or macrophage-depleted (clodronate liposomes) mice (n = 6). Mice were administered daily oral gavage of osimertinib (5 mg/kg). In parallel, intraperitoneal injections of Anti-SAA1 (0.25 μg/g) or control IgG were given every three days. Macrophage depletion was initiated on day 18 post-tumor engraftment via injections of clodronate liposomes or control liposomes (200 μL), followed by maintenance injections (200 μL) every three days (*n* = 6). K Tumor weight of PDX models treated with control therapy, Anti-SAA1, osimertinib, or the combination (*n* = 3 for each PDX). Scale bar, 50 μm. Dunnett’s multiple comparison tests were applied for panels A and K; Student’s t-test was applied for panels B, F, and H, and two-way ANOVA was applied for panel J. Abbreviations: PR, parental; OR, osimertinib resistant; CDX, cell-derived xenografts; H&E, hematoxylin and eosin; PDX, patient-derived xenografts.
